# Genetic lineage tracing defines myofibroblast origin and function in the injured heart

**DOI:** 10.1038/ncomms12260

**Published:** 2016-07-22

**Authors:** Onur Kanisicak, Hadi Khalil, Malina J. Ivey, Jason Karch, Bryan D. Maliken, Robert N. Correll, Matthew J. Brody, Suh-Chin J. Lin, Bruce J. Aronow, Michelle D. Tallquist, Jeffery D. Molkentin

**Affiliations:** 1 Department of Pediatrics and Molecular Cardiovascular Biology, Cincinnati Children's Hospital Medical Center, Cincinnati, Ohio 45229, USA; 2 Center for Cardiovascular Research, John A. Burns School of Medicine, University of Hawaii, Honolulu, Hawaii 96813, USA; 3 Howard Hughes Medical Institute, Cincinnati Children's Hospital Medical Center, Cincinnati, Ohio 45229, USA

## Abstract

Cardiac fibroblasts convert to myofibroblasts with injury to mediate healing after acute myocardial infarction (MI) and to mediate long-standing fibrosis with chronic disease. Myofibroblasts remain a poorly defined cell type in terms of their origins and functional effects *in vivo*. Here we generate *Postn* (periostin) gene-targeted mice containing a tamoxifen-inducible Cre for cellular lineage-tracing analysis. This *Postn* allele identifies essentially all myofibroblasts within the heart and multiple other tissues. Lineage tracing with four additional Cre-expressing mouse lines shows that periostin-expressing myofibroblasts in the heart derive from tissue-resident fibroblasts of the Tcf21 lineage, but not endothelial, immune/myeloid or smooth muscle cells. Deletion of periostin^+^ myofibroblasts reduces collagen production and scar formation after MI. Periostin-traced myofibroblasts also revert back to a less-activated state upon injury resolution. Our results define the myofibroblast as a periostin-expressing cell type necessary for adaptive healing and fibrosis in the heart, which arises from Tcf21^+^ tissue-resident fibroblasts.

## Abstract (web summary)

The origin and fate of myofibroblasts, the cells responsible for cardiac remodelling and fibrosis, is controversial. Here the authors show that cardiac myofibroblasts express periostin, derive exclusively from tissue-resident fibroblasts, are necessary for scar formation after injury, and can revert back to a less-activated state upon injury resolution.

## Graphical abstract

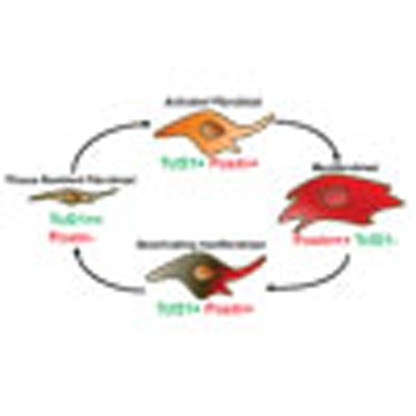

Heart disease remains the number one cause of mortality in the Western world, with myocardial infarction (MI)-based injury and subsequent ventricular remodelling and heart failure as the major sequela underlying this lethality^1^. During MI, a portion of viable myocardium is lost and immediately replaced with a fibrotic scar that prevents ventricular wall rupture. In long-standing heart failure, interstitial fibrosis accumulates and leads to a restrictive cardiomyopathy with worsening cardiac function^2^. Both types of fibrotic responses result in the activation of fibroblasts into a cell type known as the myofibroblast, which mediates extracellular matrix (ECM) production and tissue remodelling through the inherent contractile activity of these cells^3^. The myofibroblast arises from the transdifferentiation of a number of potentially different cell sources within the injured heart, although the exact precursor cell type remains an area of ongoing controversy^4^. The formation of myofibroblasts is mediated by an increase in wall tension and/or cytokine signalling^2^,^5^.

The heart becomes populated with fibroblasts during embryonic development from epicardial and endothelial derived cells that invade the heart^6^. A majority of these cells develop from transcription factor 21 (Tcf21) (ref. 7), Wilms tumour 1 (Wt1) (ref. 8) or T-box 18 (Tbx18) (ref. 9) expressing lineages, although only Tcf21 continues to be expressed within resting fibroblasts of the adult heart^10^. During development, Wt1 lineage-traced fibroblasts contribute to 80–85% of the myofibroblasts within the left ventricle of the adult mouse heart after pressure overload stimulation^11^. However, many other cell types have been suggested as a major source for newly converted myofibroblasts within the diseased mouse heart. Specifically, endothelial-to-mesenchymal transition of resident endothelial cells was proposed to generate 70% of the myofibroblasts in the heart with pressure overload^12^,^13^. Pericytes, which are cells surrounding the vasculature, were also reported to be a major source for newly generated smooth muscle α-actin (αSMA) expressing myofibroblasts in the heart^14^. Finally, bone marrow-derived myeloid cells, fibrocytes and other infiltrating immune cells have been suggested to generate myofibroblasts in the injured heart^15^,^16^,^17^. Hence, the cellular origin of the cardiac myofibroblast remains unresolved.

Two significant issues have contributed to the discordant studies discussed above. One is the lack of an appropriate marker to uniformly identify resident fibroblasts and myofibroblasts within the heart. With respect to this issue, most previous analyses were based on co-labelling with panels of antibodies, none of which were exclusive for either resident fibroblasts or myofibroblasts. Initial markers, such as thymocyte differentiation antigen 1 (Thy-1, also called CD90)^18^ and fibroblast specific protein 1 (FSP1, also called S100A4)^19^ are not specific and each labels endothelial cells, immune cells, pericytes and select other cell types^20^,^21^. More recently, platelet-derived growth factor receptor-α (PDGFRα) has emerged as a marker for fibroblasts in the heart^11^,^22^,^23^, which along with a collagen1a1-GFP expressing transgene^7^,^11^,^23^,^24^, appear to identify the majority of resident fibroblasts, although how these markers account for myofibroblasts in the heart remains undefined^7^,^11^. Another means of identifying resident fibroblasts in the heart is the combination of vimentin antibody positivity but exclusion of CD31 and CD45 antibody reactivity (the latter of which identify endothelial cells and myeloid cells, respectively)^25^. Finally, αSMA is a myofibroblast marker used in many previous studies^26^, although it is also expressed in smooth muscle cells and antibody-based strategies to detect this protein within cells of the heart are often difficult to interpret. A second issue that has caused confusion in the field is that select Cre-expressing transgenes and knock-in alleles used for fibroblast lineage tracing in the past often lacked proper specificity or showed expression in unanticipated cell types^4^.

Periostin is another described marker of the myofibroblast that is expressed in adult tissues only after injury^27^. Periostin is a secreted matricellular protein involved in cellular adhesion and organization of collagen. Deletion of the *Postn* gene in mice renders cardiac fibroblasts unable to fully function and generate a proper scar after infarction injury, although heterozygous mice are normal^28^,^29^.

Here we generate mice containing a tamoxifen-inducible Cre recombinase (MerCreMer) expression cassette within the *Postn* genetic locus (*Postn*^*MCM*^). Using these knock-in mice, we show that the *Postn* genetic locus exclusively marks essentially all cardiac myofibroblasts without ectopic expression in other cardiac cell types. Lineage-tracing analyses with four additional Cre-expressing mouse lines show that nearly all of the periostin-labelled myofibroblasts in the heart arise from tissue-resident fibroblasts that express Tcf21.

## Results

### Periostin is exclusively expressed in areas of injury

**Figure 1:**
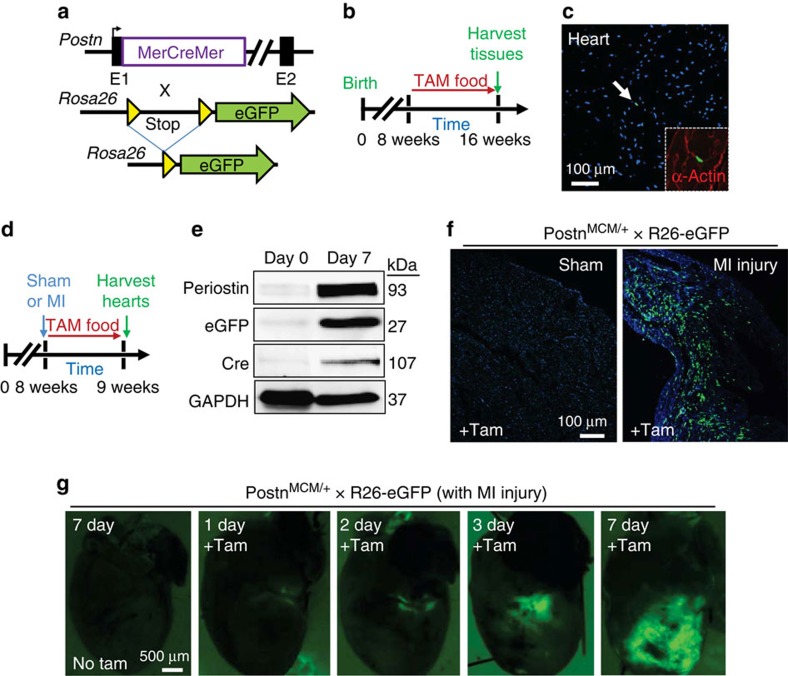
*Postn*^MCM^ allele activity *in vivo*. (**a**) Schematic representation of the *Postn* genetic locus with a tamoxifen-regulated MerCreMer cDNA cassette inserted into exon 1 (E1), which was crossed with *Rosa26* reporter mice (R26-eGFP) containing loxP sites flanking a stop cassette upstream of eGFP to allow for Cre-dependent lineage tracing. (**b**) Experimental scheme whereby *Postn*^*MCM/+*^; R26-eGFP mice were given tamoxifen for 8 weeks before harvesting at 16 weeks. (**c**) Representative histological section from the heart of mice described in **a** and **b**, which show exceptionally rare labelling of interstitial cells at baseline (arrow) with 8 weeks of tamoxifen. Nuclei are stained in blue. Inset shows α-actin stained cardiomyocytes (red) surrounding the one eGFP-labelled interstitial cell (green) (*n*=4 mice). (**d**) Experimental scheme whereby *Postn*^*MCM/+*^; R26-eGFP mice were MI injured or subjected to a sham procedure, then given tamoxifen for 1 week before harvesting. (**e**) Western blot analysis for periostin, eGFP, Cre (MerCreMer protein) and GAPDH as a control at day 0 before injury or day 7 after MI injury with 1 week of tamoxifen (*n*=3 mice per condition). (**f**) Representative histological sections showing eGFP-labelled interstitial cells in hearts of *Postn*^*MCM/+*^; R26-eGFP after MI injury with 7 days of tamoxifen labelling, but not with a sham procedure (*n*=6 mice for MI and *n*=3 for sham). (**g**) Whole-mount fluorescent images of hearts from *Postn*^*MCM/+*^; R26-eGFP mice for direct eGFP fluorescence over the given time course shown. A no tamoxifen control 7 days after MI is also shown (*n*=3 mice per time point and condition).

While periostin is expressed almost exclusively in myofibroblasts in areas of tissue injury, it is a secreted protein and therefore cannot be used to identify cell types with an antibody-based approach. Hence, we generated a *Postn* knock-in mouse line containing the MerCreMer cDNA cassette (*Postn*^*MCM*^) for tamoxifen-regulated Cre activity (Fig. 1a and Supplementary Fig. 1). These mice were bred with a *Rosa26* (R26) loxP-inactivated eGFP (R26-eGFP)^30^ reporter line for experimental lineage tracing of cells (Fig. 1a). When these two alleles are crossed, any cell that expresses periostin in the presence of tamoxifen will permanently express eGFP. To first assess both the potential for nonspecific expression and leakiness of the system, *Postn*^*MCM/+*^; R26-eGFP mice were fed tamoxifen for 8 weeks, beginning at 8 weeks of age, but without injury (Fig. 1b). The data show <1% of interstitial cellular labelling in uninjured heart, skeletal muscle, kidney, lung, liver and skin (Fig. 1c and Supplementary Fig. 2a).

With acute MI injury and 1 week of tamoxifen labelling (Fig. 1d), *Postn*^*MCM/+*^; R26-eGFP mice showed abundant expression of periostin protein in the heart, the MerCreMer protein and the recombined eGFP protein from the *Rosa26* locus (Fig. 1e). At the histological level, sham-operated mice treated with tamoxifen showed no eGFP^+^ interstitial cells in the heart, while 7 days after MI injury these mice had abundant eGFP^+^ interstitial cells in the left ventricle within the infarct region only (Fig. 1f). Moreover, acute injury to lungs, skeletal muscle or skin also showed abundant induction of eGFP^+^ interstitial cells for the first time (Supplementary Fig. 2b,c). Finally, a time course after MI injury was performed by whole-mount imaging of hearts from *Postn*^*MCM/+*^; R26-eGFP mice. Mice were given tamoxifen the entire time and imaged at 1, 2, 3 or 7 days after MI, which showed specific and progressive eGFP labelling of only interstitial cells within the heart, but never myocytes themselves, and at the infarction injury site, starting as early as 1 and 2 days (Fig. 1g).

### Periostin expression is restricted to myofibroblasts

**Figure 2:**
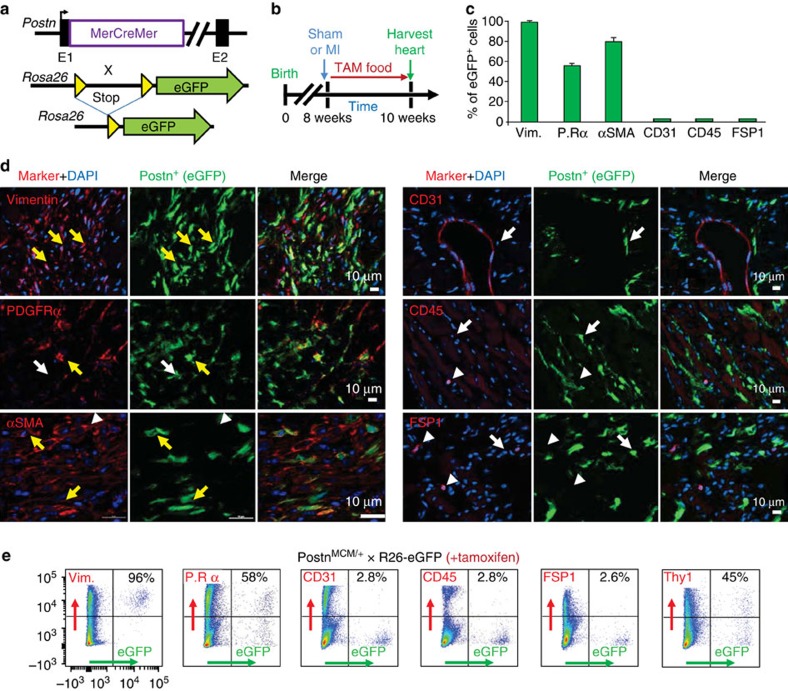
*Postn*^MCM^ allele labels myofibroblasts after MI injury. (**a**) Schematic representation of the *Postn*^MCM^ mouse crossed with a *Rosa26-eGFP* reporter mouse (R26-eGFP) for lineage tracing. (**b**) Experimental scheme to lineage trace periostin-expressing cells *in vivo* for 2 weeks with tamoxifen treatment immediately after MI injury or a sham procedure. (**c**) Quantification of co-labelling of eGFP^+^ (periostin^+^) cells with cell markers from immunohistochemical processed heart sections. Data are averaged from three hearts with >20 sections each quantified. P.Rα signifies PDGFRα. (**d**) Representative immunohistochemical images for eGFP cellular expression (green) of periostin^+^ cells and co-staining for vimentin, PDGFRα, αSMA, CD31, CD45 or FSP1 in red. The yellow arrows show co-staining, the white arrows show eGFP/periostin^+^ only, and the white arrow heads show marker expression only without eGFP^+^. (**e**) Representative flow cytometry plots of isolated eGFP^+^ cells (rightward scatter) against the cell markers depicted (upwards scatter). The percentage of cells that are marker^+^ among the GFP^+^ population of cells is shown in each upper right quadrant of the individual FACS plots and was averaged from four hearts each. All error bars represent s.e.m.

Next, the identity of the interstitial cell population labelled in *Postn*^*MCM/+*^; R26-eGFP mice was interrogated after MI injury and 2 weeks of tamoxifen treatment (Fig. 2a,b). Hearts were harvested and serial cryosections were processed to show fibrosis and coincident eGFP^+^ cell labelling (periostin lineage-traced) along with antibodies against vimentin, PDGFRα, αSMA, CD31, CD45 and FSP1 to identify fibroblasts or other interstitial cell types (Fig. 2c,d). Approximately 98% of the periostin lineage-traced cells were vimentin positive, while more than half were PDGFRα-positive and ∼80% were αSMA-positive but almost none were CD31, CD45 or FSP1 reactive (Fig. 2c,d). Consistent with these results, florescent-activated cell sorting (FACS) analysis of enzymatically isolated eGFP^+^ cells from injured *Postn*^*MCM/+*^; R26-eGFP mouse hearts quantitatively confirmed the histological results, with nearly an identical marker profile (Fig. 2e). Thy1 was also uniquely used in the FACS analysis since it is a surface marker and it has been reported to identify fibroblasts^31^,^32^ (Fig. 2e). Since the presence of vimentin reactivity and absence of CD31 and CD45 reactivity is a criterion for total fibroblast identity, and given that αSMA also marks myofibroblasts, our results indicate that periostin lineage-traced cells are myofibroblasts in the injured heart (more evidence is shown below). PDGFRα is also an accepted surface marker for resident fibroblasts in the heart^7^,^23^, which our analysis showed ∼54–58% concordance with periostin lineage-traced myofibroblasts (Fig. 2c,e). However, PDGFRα mRNA expression is downregulated in myofibroblasts compared with inactive, tissue-resident fibroblasts that uniformly express this marker (discussed below).

A recent study suggested that the type of cardiac injury might induce different populations of cells to become myofibroblasts^10^. Hence, in addition to MI injury we also performed cardiac pressure overload by transverse aortic constriction (TAC) and infusion of the profibrotic neuroendocrine agonists, angiotensin II and phenylephrine (Ang/PE), as additional models of cardiac fibrosis. Both disease stimulations were conducted for 2 weeks in *Postn*^*MCM/+*^; R26-eGFP mice, concurrent with tamoxifen treatment (Supplementary Figs 3a,b and 4a,b). Both stimuli generated a large induction of periostin lineage-traced eGFP^+^ cells throughout the heart (Supplementary Figs 3c and 4c), which by immunohistochemistry were again defined as myofibroblasts (Supplementary Figs 3d and 4d). One unique aspect of the pressure overload response is that eGFP^+^ cells were concentrated within the left ventricular free wall, septum and left atria, regions of the heart that are particularly stretched in response to pressure overload, but less so in the right ventricle and right atria (Supplementary Fig. 3c). In contrast, eGFP^+^ cellular distribution was more uniform throughout the entire heart with 2 weeks of Ang/PE stimulation (Supplementary Fig. 4c). Taken together, these data suggest that periostin lineage-tracing identifies myofibroblasts in response to a wide range of cardiac stimuli that invoke a fibrotic response.

### Periostin-labelled fibroblasts are functional in disease

**Figure 3:**
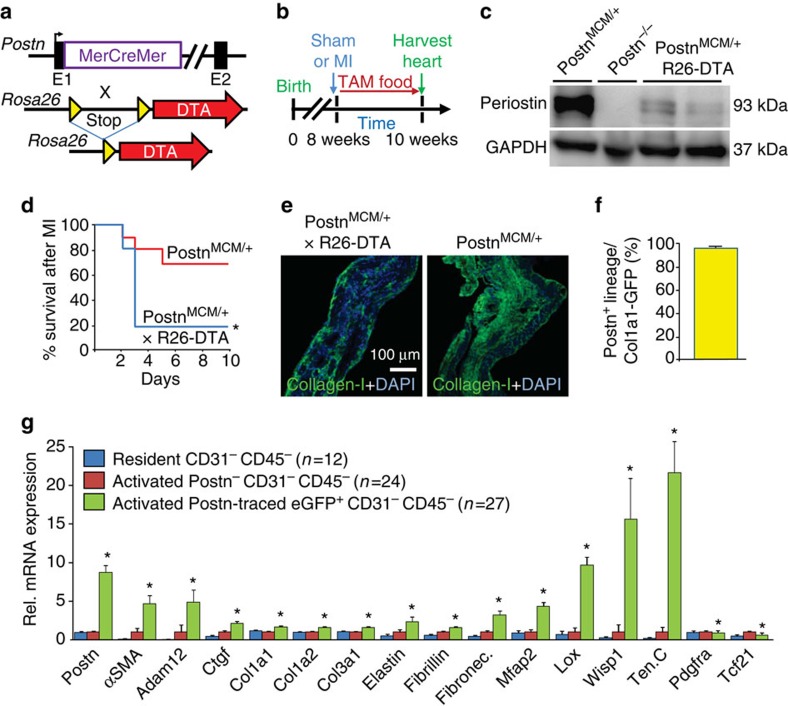
Periostin^+^ interstitial cells mediate cardiac fibrosis. (**a**) Schematic representation of the *Postn*^MCM^ mouse crossed with *Rosa26*-DTA mouse to permit killing of all periostin^+^ cells. (**b**) Experimental scheme to delete all periostin-expressing cells over 2 weeks of cardiac remodelling after MI injury when tamoxifen is present to induce MerCreMer protein activity. (**c**) Western blot analysis from the hearts of *Postn*^*MCM/+*^; R26-DTA mice 7 days after MI injury from the genotypes shown. Periostin and GAPDH (control) protein expression are shown. Heart-protein extracts from a *Postn*^*−/−*^ mouse is shown as a negative control, and a heterozygous *Postn*^*MCM/+*^ mouse is the positive control for endogenous periostin expression with injury. (**d**) Survival plot in days following MI injury for the two genotypes of mice shown, both treated with tamoxifen for 2 weeks (*n*=18 for *Postn*^*MCM/+*^; R26-DTA and *n*=12 *Postn*^*MCM/+*^, *P<0.05 versus *Postn*^*MCM/+*^). (**e**) Collagen type 1 immunohistochemistry (green) from the infarct region of the heart from mice shown in **d**. Nuclei are shown in blue. (**f**) Quantification of periostin lineage-traced cells from the heart after MI injury, which are also positive for current Col1a1-GFP expression. Data are averaged from three hearts with greater than three non-consecutive entire heart sections fully quantified. (**g**) Quantification of the indicated mRNAs in the defined cell populations shown, from hearts of *Postn*^*MCM/+*^; R26-eGFP mice 7 days after MI injury. CD31 (endothelial) and CD45 (myeloid) cells were excluded, and then eGFP^+^ or eGFP^−^ cells were collected for single-cell RNAseq. ‘Activated’ cells were generated from the infarct region directly, while resident cells were from non-MI injured hearts. *P<0.05 versus non-Postn cells in the infarct region that were also CD31^−^ CD45^−^. Number of cells analysed is shown in the graph. Activated Postn^+^ CD31^−^ CD45^−^ cells were visualized to confirm myofibroblast features. All error bars in the figure represent s.e.m. For statistical analyses student’s two-tailed *t*-test have been performed.

To assess the relevance of periostin-labelled myofibroblasts during cardiac injury and fibrosis, we generated compound heterozygote mice carrying the *Postn*^*MCM*^ allele and the *Rosa26*-DTA (diphtheria toxin A) allele, which generates an inducible Cre-dependent means of killing cells *in vivo* (Fig. 3a). Here *Postn*^*MCM/+*^; R26-DTA mice were given tamoxifen continuously after MI surgery and hearts from surviving mice were harvested 2 weeks afterwards (Fig. 3b). Ablation of periostin^+^ cells was verified by western blot, which showed a dramatic reduction in periostin protein in hearts of *Postn*^*MCM/+*^; R26-DTA mice after tamoxifen, compared with *Postn*^*MCM*^ control mice not containing the R26-DTA allele (Fig. 3c). *Postn*^*−/−*^ heart-protein extract was used as a control. Most importantly, *Postn*^*MCM/+*^; R26-DTA mice subjected to MI injury showed much greater lethality in the first few days due to ventricular wall rupture, which is consistent with lethality in *Postn*^*−/−*^ mice subject to the same injury due to a defect in the formation of a protective scar, or in a subset of developing embryos when collagen-dependent structural regions are formed in the cardiovascular system in and around the heart (Fig. 3d)^28^,^33^. Indeed, the few *Postn*^*MCM/+*^; R26-DTA mice that survived 14 days after MI injury showed reduced collagen levels in the infarct area (Fig. 3e). These results suggest that periostin-expressing myofibroblasts are required to generate a protective scar after MI injury.

### Periostin lineage-tracing labels all myofibroblasts

To more definitively examine the extent to which the periostin^+^ cells account for all of the collagen-producing myofibroblasts in the heart we also crossed the lineage-tracing *Postn*^*MCM/+*^; R26-tdTomato mouse with transgenic mice expressing GFP under the control of the collagen1a1 chimeric promoter (Supplementary Fig. 5a). The collagen1a1 promoter used to make the GFP transgene is a composite of a proximal promoter and an upstream DNase I hypersensitivity (HS4,5) region that conveys unique properties to the transgene, such that GFP is only expressed in tissue-resident fibroblasts and myofibroblasts but no other cell types^7^,^11^,^23^,^24^ (Supplementary Fig. 5a). The data show that nearly all periostin lineage-traced cells analysed 7 days post-MI in the injury region of the heart were collagen1a1-GFP expressing (Fig. 3f and Supplementary Fig. 5b,c). To further determine if periostin lineage-traced cells from *Postn*^*MCM/+*^; R26-eGFP mice accounted for all myofibroblasts in the heart we performed single-cell sorting by Fluidigm followed by RNAseq analysis after MI injury, versus uninjured hearts. Analysis of the transcriptome from 185 individual cells that passed quality assurance (see Methods) was performed from the hearts of MI injured and sham mice, and 63 of these specifically sorted into the following non-myocyte interstitial cell groups that were analysed here: (1) resident interstitial cells from uninjured hearts that are eGFP^−^ CD31^−^ CD45^−^; (2) interstitial cells from the infarct region (activated) that are eGFP^−^ CD31^−^ CD45^−^; and (3) interstitial cells from the infarct region (activated) that are eGFP^+^ (periostin lineage-traced) CD31^−^ CD45^−^. The bioinformatics analysis showed that the eGFP^+^ cells have a gene expression signature of a myofibroblast, with expression of essentially all hallmark ECM genes and ECM processing genes (Fig. 3g and Supplementary Data 1 and Supplementary Fig. 5d). Importantly, periostin-negative, non-GFP cells from the infarct region, which were excluded for CD31 and CD45 positivity, had essentially no gene signature associated with the myofibroblast, similar to non-fibroblast mesenchymal cells from uninjured hearts (Fig. 3g). As will be discussed later, collagen expression appears to extend to many other cell types and not just myofibroblasts (see below). In conclusion, these results indicate that the remaining *Postn*-negative mesenchymal cells from the infract injury region of the heart (eGFP^−^) lack a gene profile consistent with the myofibroblast, suggesting that the periostin lineage accounts for the vast majority if not all myofibroblasts generated in the heart after MI injury.

**Figure 4:**
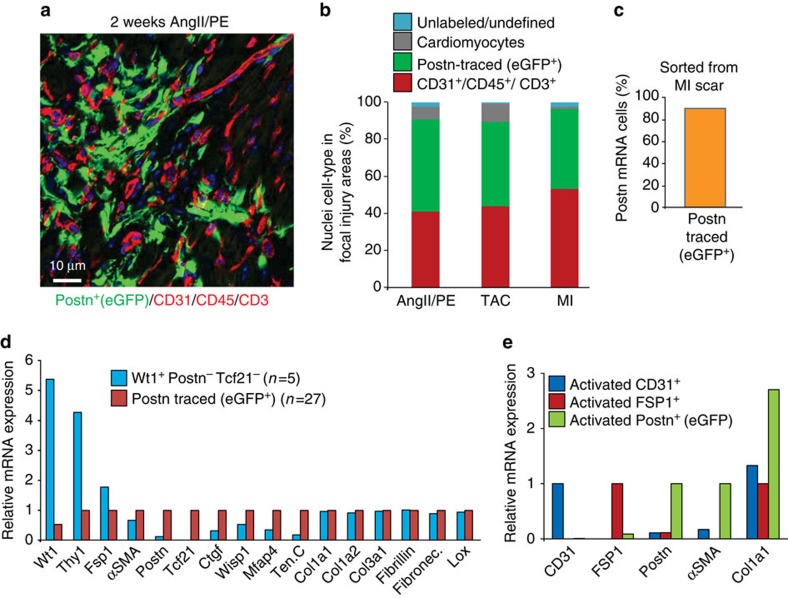
Periostin lineage tracing accounts for essentially all the myofibroblasts in the heart. (**a**) Representative histological image of an area of focal fibrosis in the hearts of *Postn*^*MCM/+*^; R26-eGFP mice 2 weeks after Ang/PE infusion. Immunohistochemistry is shown for CD31/CD45/CD3 in red, myofibroblasts are shown in green (periostin lineage traced), and myocytes can be quantified based on autofluorescence imaging. All cellular staining was matched to nuclei (blue), which (**b**) generated a quantitative assessment of the total cellularity in the Ang/PE focal injury areas, or focal injury areas with TAC and MI surgery (2 weeks afterwards). Data were averaged from three hearts for each condition with greater than five non-consecutive sections containing at least three infarct zones quantified. (**c**) Quantification of Postn-traced (eGFP^+^) cells within Postn mRNA expressing CD31^−^CD45^−^ cells isolated from the infarct region of *Postn*^*MCM/+*^; R26-eGFP mice generated from single-cell RNAseq analysis. The data show recombination efficiency of the *Postn*^*MCM*^ allele. (**d**) Relative mRNA expression for the indicated genes generated from single-cell RNAseq analysis of 152 cells isolated from the MI injury region, from which 27 were selected as perisotin lineage-traced (eGFP^+^) myofibroblasts and 5 were averaged as a unique cell type that had an unorthodox fibroblast-like profile and negative for eGFP (*Postn*). (**e**) Relative mRNA expression for the genes shown from cells sorted from the MI region (‘activated’) of the heart for CD31 expression, FSP1 expression or periostin expression (eGFP^+^).

To further validate this conclusion, we also conducted an extensive histology subtractive-processing approach from areas of focal injury and fibrosis to the heart after AngII/PE infusion, TAC and MI injury in *Postn*^*MCM/+*^; R26-eGFP mice with continuous tamoxifen administration. The percentage of nuclei-tracked cells (blue nuclear staining) was assessed by immunohistochemistry for the combination of CD31/CD45/CD3 as the red channel, eGFP (green) for periostin lineage-traced myofibroblasts, and autofluorescence shadow imaging of cardiomyocytes (Fig. 4a,b). Using this subtractive histological strategy, nuclei from cells that were unlabelled in these focal injury areas might represent other sources of fibroblasts, or simply other mesenchymal cells of unknown aetiology. Remarkably, <3% of the cells in focal fibrotic areas of the left ventricle and septum were unaccounted for, and hence could represent a minor cell population with potential myofibroblast-like identity that was not periostin lineage traced (Fig. 4b). As a technical consideration, a percentage of unidentified cells could still have been bonafide periostin-expressing myofibroblasts, but were simply missed by the MerCreMer-based lineage-tracing strategy as it was only 90% penetrant when compared against periostin mRNA expressing cells from the injury area (Fig. 4c).

While incomplete efficiency likely accounts for some of the unidentified cells in the focal fibrotic areas of the injured heart, we further analysed 152 individual cells from the MI region of the heart using Fluidigm sorting and RNAseq profiling (this number is from the 185 total cells sequenced, 33 of which were from sham hearts). We first systematically analysed the data for collagen-expressing cells that were also negative for our lineage-tracing gene, Postn, and identified five cells that had an mRNA signature of a peculiar fibroblast-like cell type (Fig. 4d). Compared with the periostin lineage-traced cells, these five cells were strongly Wt1 and Thy1 expressing, intermediate for FSP1 and αSMA, yet mostly negative for periostin and Tcf21 (Fig. 4d). However, these cells weakly expressed other known myofibroblast genes such as tenascin C, connective tissue growth factor (Ctgf) and microfibrillar-associated protein 4 (Mfap4), although they did express various collagens, Lox, fibronectin, or fibrillin compared with periostin^+^ Tcf21^+^ myofibroblasts (Fig. 4d). Collagen1a1, 1a2 and 3a1 are also expressed by many other parenchymal cell types, not just fibroblasts or myofibroblasts (Fig. 4e and Supplementary Fig. 6)^34^. Indeed, while periostin-traced myofibroblasts expressed the highest levels of collagen1a1 mRNA, CD31^+^ and FSP1^+^ sorted cells from the focal injury areas of the heart also showed expression of this gene (Fig. 4e). We also observed that cardiomyocytes from the heart express collagen1a1 mRNA in an injury inducible manner, although fivefold lower than levels of expression observed in a myofibroblast (Supplementary Fig. 6). In conclusion, periostin expression and lineage tracing with the *Postn*^*MCM*^ allele appears to account for essentially all of the myofibroblasts in the injured heart based on the expression of known marker genes, although collagen mRNA was not a reliable means of fibroblast identification.

### Origins of periostin-labelled adult cardiac fibroblasts

**Figure 5:**
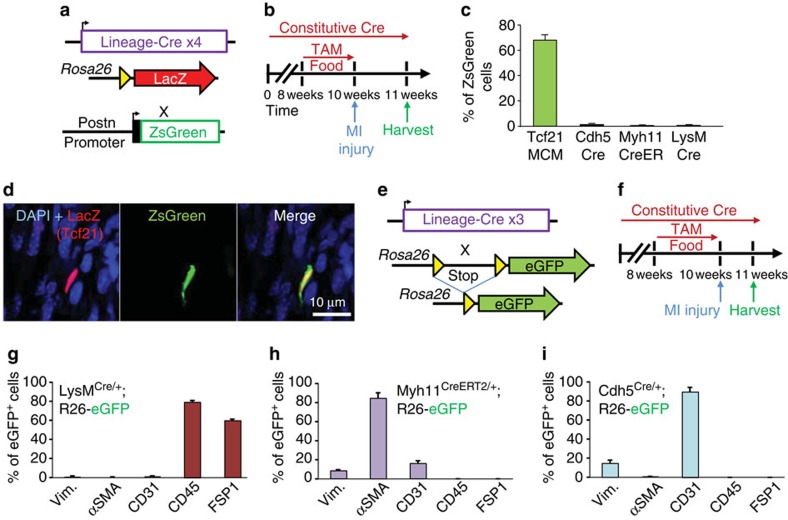
Periostin^+^ myofibroblasts are derived from the Tcf21 lineage. (**a**) Schematic representation of four different lineage-specific Cre-expressing mouse lines crossed with a LacZ-expressing reporter in the *Rosa26* locus, further crossed with mice containing a periostin promoter transgene-driving ZsGreen. (**b**) Experimental scheme to lineage trace from each of four different Cre-expressing mouse lines at baseline and after MI injury, harvested 1 week later. The Myh11^CreERT2/+^ mice required tamoxifen treatment for 2 weeks before MI injury to generate traced cells, and the tamoxifen was removed 3 days before MI surgery. (**c**,**d**) Quantification and representative images of lineage-traced cells (red, for LacZ) and ZsGreen from the periostin transgene from the MI region of the heart. LacZ was detected with an antibody (*n*=4–6 hearts, >20 sections each were quantified with >100 total ZsGreen^+^ cells counted). (**e**) Schematic representation of three different Cre-expressing knock-in mouse lines shown in **g**–**i** crossed with the eGFP expressing reporter in the *Rosa26* locus. (**f**) Experimental scheme to lineage trace from each of three different Cre-expressing mouse lines shown in **g**–**i** at baseline and after MI injury. (**g**–**i**) Quantification of immunohistochemistry analysis for vimentin, αSMA, CD31, CD45 and FSP1 that also co-labelled as lineage-traced cells from *LysM*^*Cre*^, *Myh11*^*CreERT2*^ and *Cdh5*^*Cre*^ alleles (*n*=3 hearts, >20 sections were quantified, *n*>200 cells counted for each of the indicated genotypes). All error bars in the figure represent s.e.m.

Myofibroblasts in the injured or diseased heart have been suggested to originate and transdifferentiate from many cellular sources, such as an endothelial cells, immune cells, smooth muscle cells, pericytes or epicardial derived resident fibroblasts^7^,^11^,^12^,^14^,^15^. However, there is disagreement even amongst these studies each claiming that one of these cell sources is dominant. Here we attempted to quantify the cellular sources for periostin-traced myofibroblasts in the mouse heart using lineage tracing with four independent genetic loci together with concurrent periostin expression (Fig. 5a). We used *Rosa26*^nLacZ^ reporter mice carrying either *Tcf21*^MCM^ (resident fibroblasts)^7^, *LysM*^Cre^ (macrophages)^35^, *Cdh5*^Cre^ (endothelial cells)^36^ and *Myh11*^CreERT2^ (smooth muscle cells)^37^ along with a periostin promoter transgene-driving ZsGreen^38^ (Fig. 5a). For the lineage-tracing component, mice with inducible Cre alleles were given tamoxifen for 2 weeks, then given MI injury 3 days later, while the two other mouse lines had constitutive and non-regulated Cre alleles and thus had continuous labelling (Fig. 5b). Hearts were processed for antibody detection of nuclear localized LacZ (β-galactosidase) versus ZsGreen expression from the periostin promoter^38^. The data demonstrate that nearly 70% of the currently expressing ZsGreen expressing cells were *Tcf21* lineage traced, but <1% were from the endothelial (*Cdh5*^Cre^), smooth muscle (*Myh11*^CreERT2^) or monocyte and macrophage (*LysM*^Cre^) lineages (Fig. 5c,d).

As yet another criteria for determining the extent to which select cellular origins contribute to myofibroblasts in the heart, the lineage-tracing analyses with these same Cre lines was compared against a full analysis of antibody markers as presented earlier (Fig. 5e). Mice were given tamoxifen food for 2 weeks, allowed 3 days off, then infarcted at 10 weeks of age and harvested 1 week later (Fig. 5f). *LysM*^Cre^ labelled cells primarily gave rise to CD45 and FSP1 expressing cells, but they lacked markers of fibroblasts (Fig. 5g and Supplementary Fig. 7). The *Myh11*^CreERT2^ traced cells lacked vimentin and histological analysis showed a localization pattern to the media of vessels in the heart where smooth muscle cells reside, but not within the infarct region that would be characteristic of myofibroblasts (Fig. 5h and Supplementary Fig. 7). Endothelial cells labelled with the *Cdh5*^Cre^ allele were mostly CD31 positive and only a small portion were co-labelled for vimentin, while none were αSMA expressing further suggesting that endogenous endothelial cell lineages do not generate myofibroblasts in the heart with MI injury (Fig. 5i and Supplementary Fig. 7). Thus, endothelial cells, smooth muscle cells and immune cells are negligible sources for generating myofibroblasts in the MI-injured adult mouse heart.

Since resident Tcf21 lineage-traced cells were the overwhelming source of periostin-expressing myofibroblasts in the infarct region of the heart, a more elaborate investigation of Tcf21 expression and lineage-traced cells was undertaken using *Tcf21*^*MCM/+*^; R26-eGFP mice (Supplementary Fig. 8a). Here, we first began with uninjured mice since Tcf21 is highly expressed in tissue-resident fibroblasts at baseline within the heart^7^,^10^,^39^. *Tcf21*^*MCM/+*^; R26-eGFP mice were given tamoxifen for 2 weeks and then harvested (Supplementary Fig. 8b). The lineage-tracing strategy labelled large numbers of resident fibroblasts throughout the uninjured adult heart, which were positive for vimentin and PDGFRα, but not αSMA, CD31 or FSP1 (Supplementary Fig. 8c,d). These results were confirmed by quantitative FACS analysis, which again showed that all resting Tcf21 lineage-traced fibroblasts from the heart expressed vimentin but not CD31, CD45 or FSP1 (Supplementary Fig. 8e,f).

**Figure 6:**
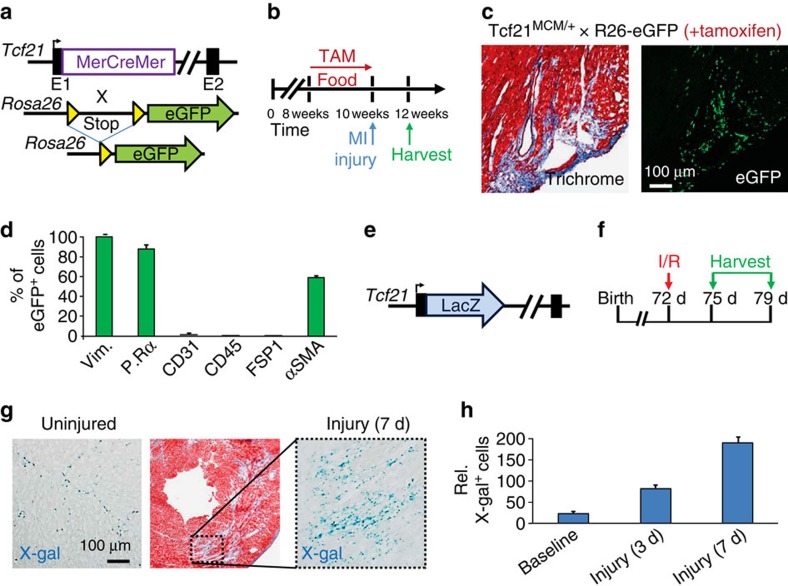
Tcf21^+^ resident fibroblasts in the adult heart expand with injury. (**a**) Schematic representation of the *Tcf21*^MCM^ mouse crossed with a *Rosa26*-eGFP reporter mouse for lineage tracing. (**b**) Experimental scheme to lineage trace Tcf21-expressing cells *in vivo* for 2 weeks with tamoxifen until 3 days before MI injury, then hearts are harvested 2 weeks after MI injury without tamoxifen. (**c**) Representative Masson’s trichrome-stained histological section and a parallel section showing eGFP expression in the infarct and border zone region of an MI-injured heart from *Tcf21*^*MCM/+*^; R26-eGFP mice. (**d**) Quantification of Tcf21 lineage-traced (eGFP^+^) fibroblasts numbers from histological sections of hearts from *Tcf21*^*MCM/+*^; R26-eGFP mice 2 weeks after MI injury that were positive by immunohistochemistry-based analysis for the six markers shown along the bottom of the graph (*n*=3 hearts, >20 sections each were quantified for >200 cells). P.Rα signifies PDGFRα. (**e**) Schematic representation of the *Tcf21*^LacZ^ knock-in allele containing mouse, which was used (**f**) to show real-time Tcf21 expression in the heart 3 and 7 days after cardiac ischaemia/reperfusion (I/R) injury in young adults. (**g**) Histological sections from the hearts of *Tcf21*^LacZ^ mice stained for LacZ expression using x-gal as a substrate (blue) at baseline or 7 days after injury. A Masson’s trichrome-stained heart section is also shown, which stains fibrotic material in blue and normal myocardium in red. (**h**) Quantification of total LacZ-expressing cells (x-gal stained) at baseline and 3 and 7 days after injury in the focal fibrotic regions of the heart, showing expansion of Tcf21-expressing cells. Data are form *n*>3 hearts per time point with greater than five non-consecutive sections from infarct zone quantified. Error bars represent s.e.m.

Next we performed lineage tracing after MI injury in *Tcf21*^*MCM/+*^; R26-eGFP mice. Tamoxifen was given for 2 weeks before MI (with 3 days no treatment before injury), followed by harvesting of hearts 2 weeks later for analysis (Fig. 6a,b). The results showed a 10-fold increase in total Tcf21-labelled fibroblasts in the infarct region and associated border zone, reminiscent of how periostin-labelled myofibroblasts similarly expand (Fig. 6c). Immunohistochemistry-based quantification of all Tcf21 lineage-traced (eGFP^+^) fibroblasts also showed a profile consistent with periostin-labelled myofibroblasts within the infarct, in that they were positive for vimentin, αSMA and PDGFRα (Fig. 6d). To further characterize Tcf21-expressing cells in a similar mouse model of ischaemia-reperfusion (I/R) injury to the heart, a *Tcf21*^LacZ^ knock-in allele was used to mark currently expressing fibroblasts (Fig. 6e,f). An I/R model often generates a smaller injury area compared with MI so that expansion can be better examined. While the uninjured heart again showed expression in tissue-resident fibroblasts throughout the heart (corresponding to ∼10% of the total cell number in the heart), areas of direct injury with ongoing fibrosis showed a 10-fold expansion of Tcf21-expressing fibroblasts up through day 7 after injury (Fig. 6g,h). Taken together, these results suggest that Tcf21 lineage-tracing labels resident fibroblasts in the heart that expand and give rise to periostin-expressing myofibroblasts with injury.

### Tcf21^+^ fibroblasts become periostin^+^ myofibroblasts

**Figure 7:**
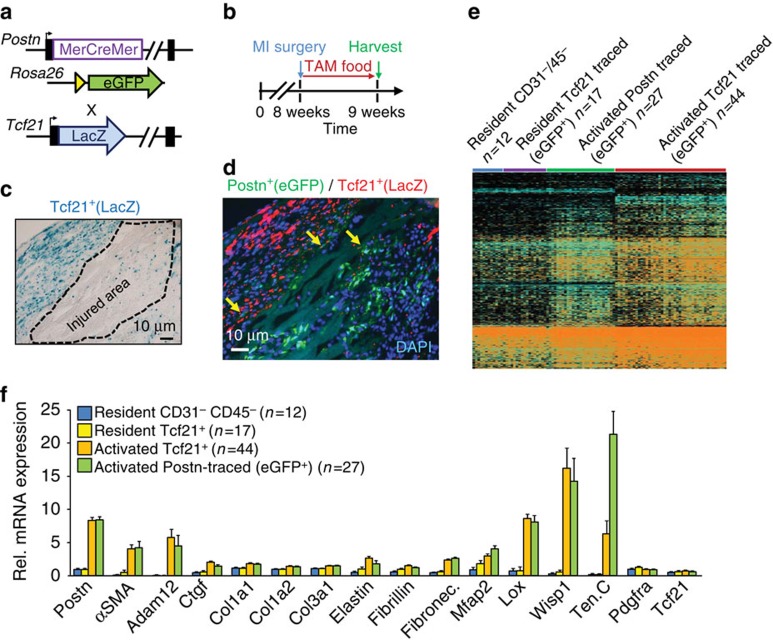
Periostin^+^ myofibroblasts derive from Tcf21 resident fibroblasts. (**a**) Schematic representation of the *Postn*^MCM^ mouse crossed with a *Rosa26*-eGFP reporter mouse (R26-eGFP) for lineage tracing, which was further crossed with the *Tcf21*^LacZ^ knock-in mouse line. (**b**) Experimental scheme to lineage trace periostin-expressing myofibroblasts *in vivo* for 1 week with tamoxifen treatment immediately after MI injury. (**c**) Representative histological section from an MI region of the heart of a *Postn*^*MCM/+*^; R26-eGFP mouse that also contained the *Tcf21*^LacZ^ allele. The section is only stained for x-gal activity (LacZ expression), and Tcf21^+^ expanded fibroblasts appear around the demarked injured region. (**d**) Same scheme as in **c** except that immunohistochemistry was used to detect LacZ (Tcf21 current expression, red staining) and periostin lineage-traced cells in green. The yellow arrows show a few rare transitional cells that express both periostin and Tcf21. Nuclei are stained in blue (*n*=4 hearts). (**e**) Thermogram of gene expression patterns from RNAseq of representative individual cells from the hearts of *Postn*^*MCM/+*^; R26-eGFP or *Tcf21*^*MCM/+*^; R26-eGFP mice. (**e**,**f**) Cells were negatively sorted for CD31 and CD45 and were either Tcf21 lineage traced (eGFP^+^) and sorted from uninjured hearts (yellow bars in **f**) or from the MI region 7 days after injury as `activated'. As another control periostin lineage-traced cells were collected from the MI region of the heart 7 days after injury for comparison. A population of total interstitial cells were used as a control, which were negatively sorted for CD31 and CD45 from the remote region of the heart. Data produced from a total of 185 cells isolated from three mice in each group in **e**, and a subset is shown in **f**. Error bars represent s.e.m.

Given the results presented above we hypothesized that Tcf21-expressing fibroblasts in the heart represented the primary progenitor pool, while expression of periostin marked progression of the same fibroblasts to myofibroblasts. To solidify this hypothesis further, *Postn*^*MCM/+*^; R26-eGFP; *Tcf21*^LacZ/+^ triple heterozygous mice were used, which allows for lineage tracing of periostin myofibroblasts and assessment of current Tcf21 expression (Fig. 7a). MI injury was also performed so as to induce periostin expression. Eight-week-old mice were subjected to MI surgery and hearts were harvested after 1 week of tamoxifen administration (Fig. 7b). Histological analysis of infarct regions showed areas of expanded Tcf21-expressing (LacZ^+^) fibroblasts within the border regions of the infarct, although some were also present within the infarct itself (Fig. 7c). However, analysis of periostin lineage-derived cells, which were highly expanded in the scar and fibrotic region of the heart, showed a loss of current Tcf21 expression (Fig. 7d). These results could suggest that Tcf21-expressing fibroblasts are more proliferative than Postn lineage-traced cells, although this issue is currently under investigation. We also observed a few rare cells that appeared to have expression of both, suggesting a transitional cell type (Fig. 7d, yellow arrows). Indeed, single-cell RNAseq analysis of 185 cells showed a gene signature whereby periostin lineage-traced cells from the infarct region expressed all the markers of myofibroblasts, while Tcf21-expressing fibroblasts from uninjured regions of the heart had an inactive profile for these same genes (Fig. 7e,f). Consistent with the immunohistochemistry, we observed that the activated Tcf21-traced cells go through an intermediate gene expression profile, such that Tcf21 was expressed along with some markers for myofibroblasts (Fig. 7e). However, the critical conclusion here is that Tcf21^+^ lineage-traced fibroblasts isolated from the infarct region become periostin-expressing myofibroblasts that are identical to periostin lineage-traced cells from the infarct region (Fig. 7f, Supplementary Data 2 and Supplementary Figs 9–11). Collectively, these results suggest that Tcf21-expressing resident fibroblasts are the primary source for generating periostin-expressing myofibroblasts in the heart with injury.

### Periostin^+^ myofibroblasts can be partially inactivated

**Figure 8:**
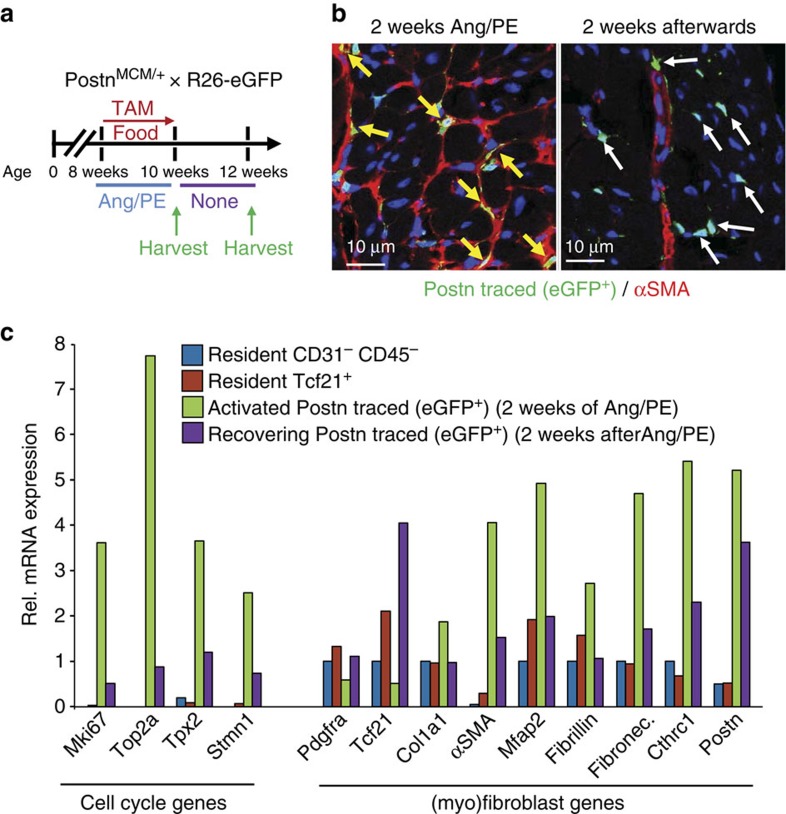
Periostin^+^ myofibroblasts can convert back towards a resident fibroblast program with cessation of tissue injury. (**a**) Schematic representation of the cardiac injury protocol used. (**b**) Representative images of immunohistochemistry for αSMA reactivity, along with eGFP^+^ cells from lineage tracing in *Postn*^*MCM/+*^; R26-eGFP mice that were given Ang/PE and tamoxifen for 2 weeks (first panel) then allowed to recover for 2 weeks with no stimulation or labelling with tamoxifen (second panel). The yellow arrows show myofibroblasts expressing αSMA (red) that were also periostin lineage-traced (green) during the injury response. The white arrows show how after regression of the fibrotic response the eGFP^+^ cells persist, but no longer express αSMA (*n*=3). (**c**) RNA expression profiling for the genes shown along the bottom of the graph, from the indicated cell types, either right after Ang/PE injury for 2 weeks, or after 2 additional weeks without stimulation. Cells were sorted as total resident mesenchymal cells lacking CD31 and CD45 from uninjured hearts (blue bars), resident Tcf21-expressing cells from uninjured hearts (red bars), activated periostin lineage-traced (eGFP^+^) myofibroblasts immediately after Ang/PE infusion (green bars), and periostin lineage-traced (eGFP^+^) cells 2 additional weeks after injury when the fibrotic response was regressing (purple bars). Data from two separate replicates pooled from three hearts for each group are shown.

The ability of periostin lineage-traced myofibroblasts to become inactivated was also analysed. Lineage tracing was performed in *Postn*^*MCM/+*^; R26-eGFP mice that were given tamoxifen to label activated fibroblasts for 2 weeks while the fibrotic agonists Ang/PE were infused with Alzet minipumps (Fig. 8a). Mice were then allowed to recover for 2 weeks with no Ang/PE as the fibrotic response regressed, and the fate of the lineage-traced eGFP^+^ cells was assessed by immunohistochemistry with concurrent αSMA immunostaining (Fig. 8a,b). The data show that immediately after 2 weeks of Ang/PE infusion nearly all the periostin lineage-traced (eGFP^+^) myofibroblasts were αSMA-positive in the heart (Fig. 8b). However, when the fibrotic response was partially regressed 2 weeks later, a number of periostin lineage-traced (eGFP^+^) cells were still present in the heart, although αSMA expression was no longer coincident (Fig. 8b). As a control, αSMA expression could still be visualized around the vasculature given the presence of smooth muscle cells (Fig. 8b).

To more carefully assess the identity of these persistent periostin lineage-traced (eGFP^+^) cells, FACS was used for cellular purification followed by RNAseq analysis (Fig. 8c). Compared with the RNAseq profile of currently activated periostin^+^ myofibroblasts taken right after 2 weeks of Ang/PE infusion, the ‘recovering’ eGFP^+^ cells showed a substantial reduction in cell cycle genes and in genes associated with the differentiated myofibroblast (Fig. 8c and Supplementary Data 3). For example, αSMA, collagen1a1, fibronectin, fibrillin, Mfap2 and Cthrc1 were all downregulated in the ‘recovering’ fibroblasts compared with myofibroblasts collected immediately after 2 weeks of Ang/PE infusion (Fig. 8c). More importantly, these ‘recovering’ periostin lineage-traced fibroblasts that remained in the heart now showed increased expression of Tcf21 and PDGFRα, both of which are downregulated in fully differentiated myofibroblasts from an active cardiac injury site (Fig. 8c). Overall, these results suggest that upon cessation of an injury response in the heart, periostin lineage-traced myofibroblasts can revert back to a state more consistent with a resident Tcf21-expressing fibroblast.

## Disscussion

The results of this study suggest a new molecular definition for the myofibroblast within the adult heart based on expression of periostin as a final common marker, which also likely applies to many other tissues and organs that can succumb to fibrotic disease under various pathological conditions. Within the heart essentially all myofibroblasts, regardless of their prior lineage, express periostin and can be directly traced using the *Postn*^*MCM*^ allele. RNAseq analysis showed that these periostin-traced myofibroblasts have a gene expression profile fully consistent with a cell type known as the myofibroblast. In addition to periostin, these cells express contractile genes such as αSMA and other genes critical for ECM production, ECM conditioning and ECM remodelling^40^. Periostin^+^ myofibroblasts from the heart, confirmed both by lineage tracing and periostin single-cell RNAseq analysis, were vimentin positive but CD31- and CD45-negative, and they also expressed the collagen1a1-GFP transgene and were intermediate positive for PDGFRα. However, PDGFRα and Tcf21 were most highly expressed in tissue-resident fibroblasts that were unstimulated, compared with lower levels of expression in periostin lineage-traced myofibroblasts. While much of the analysis was based on MI injury to the left ventricle, an identical profile was observed after pressure overload-induced cardiac hypertrophy and with Ang/PE infusion. Using a histological approach to account for all possible cell types within injury areas of the heart, in combination with single-cell RNAseq, periostin lineage-traced cells appeared to account for essentially all of the myofibroblasts present in the injured heart. This paradigm also appears to relate to all other tissues we have thus far analysed (skeletal muscle, lung and skin).

Tcf21 lineage-traced cells were shown to be the primary source for future periostin-expressing myofibroblasts in the heart after injury, with most other previously implicated lineages having either no or only a minimal contribution. Tcf21 is expressed in and marks the epicardium of the developing embryonic heart (similar to Wt1 and Tbx18), which then invades the heart as it gives rise to resident fibroblasts and smooth muscle cells. Deletion of *Tcf21* results in hearts mostly lacking fibroblasts^7^, and we previously demonstrated that Tcf21-expressing cells populate areas of cardiac fibrosis and injury in the adult heart with pressure overload and MI injury^10^. More recently, epicardial derived fibroblasts, as traced with *Wt1*^CreERT2^ or *Tbx18*^CreERT2^ alleles, were shown to give rise to 80% or more of the total fibroblasts in the left ventricle of a failing mouse heart^11^,^31^ Thus, there is strong support for the conclusion that tissue-resident fibroblasts in the adult heart are the primary cell type that generates myofibroblasts upon injury. This same paradigm appears to be present in the fibrotic cap of diseased vasculature in atherosclerotic mice where Tcf21 lineage-traced cells were shown to uniformly express αSMA, periostin and PDGFRα^41^.

The conclusion that Tcf21-expressing resident cardiac fibroblasts are the primary source of disease-activated myofibroblasts in the heart is not consistent with previous studies that have suggested alternate lineages. For example, endothelial-to-mesenchymal transition from resident endothelial cells was reported to be a major source of myofibroblasts in the heart with injury^12^,^13^. Reasons for this discrepancy may be due to the use of the constitutive Tie1^Cre^ transgenic line for lineage tracing along with αSMA and FSP1 immunohistochemistry^12^. For example, FSP1 is not specific to fibroblasts^20^, and our data suggest that FSP1 is more highly expressed in immune cells, and is largely absent in Tcf21 fibroblasts or periostin lineage-traced myofibroblasts. Moreover, the constitutive Tie1^Cre^ transgenic line is also known to be expressed in immune cells^42^. Another previous study that was discordant with our results used a constitutive Tie2^Cre^ transgenic line to track cells, although the Tie2 promoter is expressed in all hematopoietic and bone marrow cells as well^43^. Finally, cells of myeloid origin have been proposed as a major source for newly generated myofibroblasts in the heart^15^,^16^,^17^. Our results with *LysM*^Cre^ lineage tracing do not support this conclusion, and in separate studies with a *Kit*^*Cre*^ lineage-tracing system for total hematopoietic and immune cells, we also failed to observe significant myofibroblast contribution^44^.

In conclusion, the results presented here are most consistent with the hypothesis that Tcf21-expressing resident fibroblasts are the primary source of cells that directly become myofibroblasts in the heart with injury. With this new potential understanding of the cellular basis for fibrosis in the heart, it should now become more feasible to design therapies to target the activity of the periostin-expressing myofibroblast that underlies cardiac remodelling and disease responsiveness.

## Methods

### Mice

All experiments involving mice were approved by the Institutional Animal Care and Use Committee (IACUC) at Cincinnati Children’s Hospital Medical Center. Targeted *Postn*^MCM^ mice were generated by standard gene-targeting techniques. DNA homology arms upstream and downstream of the ATG start codon of the *Postn* gene were subcloned into a plasmid backbone to create a gene-targeting construct. The plasmid also contained a diphtheria toxin A (DTA) cDNA cassette for negative selection and a frt site-flanked neomycin cDNA cassette for positive selection. A cDNA encoding the MerCreMer cDNA^45^ was cloned in-frame with the *Postn* ATG start site of exon 1. Embryonic stem (ES) cells were electroporated with this linearized DNA-targeting vector and G418-resistant colonies were picked and subject to Southern blot and PCR to identify properly targeted clones. ES cell aggregation with eight-cell embryos was used to generate chimeric mice. Germline transmitting male chimeras were crossed with *Rosa26*-Flpe females (B6.129S4-*Gt(ROSA)26Sor*^*tm1(FLP1)Dym*^/RainJ) to delete the neomycin cassette at the frt sites, and verified offspring were further backcrossed to C57Bl/6J for five generations. Reporter mice FVB.Cg-*Gt(ROSA)26Sor*^*tm1(CAG*−*lacZ,EGFP)Glh*^/J (previously modified by cross-breeding to B6(C3)-Tg(Pgk1-FLPo)10Sykr/J) and B6.129(Cg)-*Gt(ROSA)26Sor*^*tm4(ACTB*−*tdTomato*,−*EGFP)Luo*^/J were purchased from the Jackson Laboratories^30^. PCR genotyping of *Postn*^MCM^ mice used the following primers, (wt-Postn-forward: 5′-TCT GTA AGG CCA TCG CAA GCT-3′; mutant-forward: 5′-GGT GGG ACA TTT GAG TTG CT-3′ and WT intron-reverse: 5′- AAT AAG TAA AAC AGC TCC CCT-3′). Other mouse lines are as follows: *LysM*^Cre^ B6N.129P2(B6)-Lyz2tm1(cre)Ifo/J] Jax stock no: ID018956; *Cdh5*^Cre^ [B6.FVB-Tg(Cdh5-cre)7Mlia/J] Jax Stock No: 006137. *Myh11*^CreERT2^ [B6.FVB-Tg(Myh11-cre/ERT2)1Soff/J] Jax Stock No:019079. Rosa26-DTA [Gt(ROSA)26Sortm1 (DTA)Jpmb/J] Jax Stock No:006331. Rosa26-nLacZ [FVB.Cg-Gt(ROSA)26Sortm1 (CAG-lacZ,-EGFP)Glh/J] Jax Stock No:012429. *Tcf21*^LacZ^ (ref. 46); *Tcf21*^MCM^ (ref. 47); collagen1a1-GFP^24^, *Postn*^*−/−*^ (ref. 28) and Postn-ZsGreen^38^ mice were previously described.

### Animal procedures

Tamoxifen citrate containing mouse chow at a treatment dosage of 400 mg kg^−1^ (Harlan laboratories) was used to activate the inducible MerCreMer protein or the CreERT2 protein, thereby inducing Cre recombinase activity. The duration of treatment is indicated within each experiment. MI was induced in mice via permanent surgical ligation of the left coronary artery^48^. Briefly, mice were anaesthetized using isoflurane and a left lateral thoracotomy was performed. The left coronary artery was identified and ligated just below the left atrium. Myocardial injury induced by I/R was used as a model with less overall injury to the myocardium, so that a more regional fibrotic response and its expansion could be examined as described previously^49^. Pressure overload by TAC is performed by tying a silk ligature around a 26-gauge wire (mice) and the transverse aorta as it leaves the heart, to generate a defined constriction when the wire is removed, which produces a pressure load on the heart leading to hypertrophy^50^. Lung fibrosis and remodelling is caused indirectly through TAC surgery and cardiac ventricular failure as described in the literature^51^. For Ang/PE treatment, micro-osmotic pumps (Azlet Model 1002) were inserted subcutaneously delivering combination of 1.5 μg g^−1^ day^−1^ angiotensin II (Sigma, A9525-50G) and 50 μg g^−1^ day^−1^ phenylephrine hydrochloride (Sigma, P6126-10G) for 2 weeks. Control animals were treated with saline. Mice were either sacrificed by CO_2_ asphyxiation or by excision of the heart under deep isoflurane sedation. Skeletal muscle injury is caused by direct injection of cardiotoxin (Sigma-Aldrich C9759-1MG) (10 μM in sterile PBS; 0.36 mg kg^−1^, 100 μl total volume) to the hind limb muscle of adult mice with a 28.5-gauge needle while mice were under mild isofluorane sedation^52^. Isolated organs were fixed in 4% paraformaldehyde (PFA) for 3.5 h, and immersed in PBS containing 30% sucrose overnight before embedding in OCT (Tissue-Tek) for cryo-sectioning. For skin injury mice received 6 mm excisional biopsy wounds created with a disposable biopsy punch apparatus (Integra Miltex) on the dorsal midline of their back under anesthesia^53^.

### Histology and immunohistochemistry

Isolated organs were fixed for 3.5 h in freshly diluted 4% PFA at 4 °C, rinsed with PBS and cryoprotected in 30% sucrose/PBS overnight before embedding in OCT (Tissue-Tek). Afterwards, 10 μm cryosections were collected and then blocked for 30 min at room temperature in a blocking solution (PBS with 5% goat serum, 2% bovine serum albumin, 0.1% Triton X-100), which was also used to dilute antibodies. The following primary antibodies were used at 1:200 dilution on cryosections: vimentin (Abcam ab45939); PDGFRα (R&D Diagnostics AF1062); αSMA (Sigma A2547); CD31 (BD Biosciences 553370); CD45 (BD Biosciences 553076); FSP1 (Abcam ab27957) and NG2 (Millipore ab5320); collagen type I (Abcam ab21286); and sarcomeric α-actin (Sigma A2172) and β-galactosidase (Abcam ab9361). Primary antibodies were incubated overnight at 4 °C. Sections were washed three times for 5 min each in PBS and incubated with a 1:500 dilution of Alexa Fluor 555-conjugated goat anti-mouse antibody (Invitrogen) in 2% BSA/5% goat serum/PBS for 45 min at room temperature. After washing three times for 5 min each, fibres were stained with DAPI and mounted on slides using aqueous mounting medium (Biomeda, Foster City, CA). Secondary antibodies were incubated for 2 h at room temperature at 1:500 dilutions, and three washes of 5 min each were performed in PBS. Cryosections were used to visualize native eGFP or tdTomato fluorescence from the different Rosa26-containing reporters. Images were acquired on an inverted Nikon A1R confocal microscope using NIS Elements AR 4.13 software. Some images were further processed in Photoshop or Image J to increase brightness/contrast of individual channels before generating a pseudo-coloured overlay. For detection of β-galactosidase (LacZ) expression adult hearts were fixed in 2% PFA in PBS (pH 7.4) for 2 h at 4 °C, and rinsed in three changes of PBS over 30 min followed by β-galactosidase staining of 10 μm sections by incubating in a solution of 2 mM MgCl_2_; 0.02% IGEPAL; 0.01% deoxycholate and 5 mM each K^+^Ferrocyanide/K^+^Ferricyanide in sodium phosphate buffer pH 7.4 containing X-Gal at 1 mg ml^−1^ concentration at 37 °C overnight. Masson’s trichrome staining was done with a kit (Sigma-Aldrich HT15-1KT) per manufacturer’s instructions. Whole heart eGFP images were captured with Leica M165FC stereo microscope with fluorescent capability using Leica DFC310 FX camera and Leica Application Suite.

### Isolation of cardiac fibroblasts

For FACS analysis whole cardiac ventricles were excised from mice, rinsed with cold sterile HBSS (Fisher Scientific, SH30588.01), and then placed in a 35 mm dishes with 300 μl DMEM (Fisher Scientific, SH30022FS) to prevent drying. For isolating fibroblasts from injury or remote regions, hearts were dissected under a stereomicroscope with fluorescence capability to precisely dissect the injury site on the left ventricle by viewing eGFP fluorescence. Ventricles or parts of the ventricles were then thoroughly minced with sterile fine scissors and digested in 10 ml of DMEM containing Worthington collagenase type 2 (LS004177) (100 U ml^−1^ or 0.2%) at 37 °C for up to 90 min total. During this incubation, the digesting tissue was triturated for a minute with a narrow-bore sterile serological pipette every 15 min. Tubes containing triturated tissue were rested vertically for 2 min and 5 ml of the unsettled supernatant cell suspension containing liberated fibroblasts was collected into a tube containing cold DMEM. The undigested fraction was brought up to 10 ml with fresh digestion media and the digestion procedure was repeated until the entire heart was liberated into single cells. After the digestion, cardiomyocytes and debris from interstitial cells were eliminated by two serial centrifugations at 10*g* for 5 min at 4 °C and the non-cardiomyocyte cell fraction was collected after a final centrifugation at 500 g for 10 min at 4 °C and pellets were resuspended in 2% fetal calf serum in HBSS. After isolation, cells were kept on ice and further processed by FACS.

### Flow cytometry and cell sorting

Flow cytometry analysis was performed on isolated cardiac interstitial cells using a BD FACSCanto II running FACSDiva software with the following configuration: 405 nm laser for Alexa405, 633 nm for APC and 488 nm for GFP. Voltages were determined using single-stain and fluorescence minus one (FMO) controls. Analysis was performed using FlowJo vX. Cells isolated as described above were either stained with surface markers using APC conjugated antibodies against CD31 (eBioscience 17-0311-82); CD45 (BD Biosciences 559864) and PDGFRα (eBioscience 17-1401-81) or with intracellular markers using unconjugated antibodies against vimentin (Abcam ab45939) and FSP1 (Abcam ab27957). For surface markers, cells were incubated for 30 min on ice with 2% fetal calf serum in HBSS containing antibodies at a 1:200 dilution. Cells were then washed three times with HBSS and analysed. For intracellular staining, cells were fixed in 4% PFA at 4 °C, and rinsed with HBSS before staining. These cells were later incubated for 30 min on ice with 2% fetal calf serum 0.1%;Triton-X 100 in HBSS containing primary antibodies at a 1:200 dilution. Cells were then washed three times and incubated for another 30 min on ice with 2% fetal calf serum 0.1%; Triton-X 100 in HBSS containing secondary antibody conjugated to Alexa flour 647 and analysed after three washes. For analysis of lineage tracing, we utilized the endogenous eGFP fluorescence expressed by the recombined reporter allele.

For FACS of lineage-traced cells, injured and uninjured regions of left ventricles were micro-dissected under dissection microscope. Total interstitial cell fractions from these injured or uninjured regions were isolated by enzymatic digestion as described above and cells were stained for surface markers of endothelial (CD31) and myeloid (CD45) populations to negatively sort and eliminate these non-fibroblast populations that would contaminate the fibroblast single-cell analyses (described below). Briefly, cells were incubated for 30 min on ice with 2% fetal calf serum in HBSS containing both CD31 (eBioscience 17-0311-82) and CD45 (BD Biosciences 559864) antibodies conjugated with APC at a 1:200 dilution. Cells were then washed three times with HBSS and 7-Aminoactinomycin D (7-AAD) (Life TechnologiesA1310), a viability dye added to the suspension before sorting. Sorting was performed with BD FACS Aria Instrument where dead cells (7AAD^+^) and non-fibroblasts (CD31^+^CD45^+^) were negatively gated before collecting either lineage-traced (eGFP^+^) or non-lineage (eGFP^−^) cells from *Postn*^*MCM/+*^; R26-eGFP or *Tcf21*^*MCM/+*^; R26-eGFP mice.

### Single-cell capture and RNA isolation

Single-cell suspensions were acquired by FACS and resuspended in HBSS, and the concentration adjusted to 350,000 cells ml^−1^. Up to 96 single cells from four separate lineage-tracing experiments were captured with the Fluidigm C1 system and the lineage positivity of the captured cells was immediately determined and mapped by fluorescent microscopy, so that cells could be classified as either eGFP-positive (Postn or Tcf21 lineage-traced) or eGFP-negative (see below for details). Single-cell RNA isolation was carried out with the Clontech UltraLow SMARTer amplification chemistry, and Illumina/Nextera tagmentation-barcoding to obtain RNA sequence from individual cells, as per Fluidigm recommended protocols. Before amplification and sequencing RNA quality was determined with an Agilent instrument with RIN (RNA Integrity Numbers) ranging from 9.2–10 with bioanalyser traces showing both the 18S and 28S ribosomal peaks present with minimal degradation were proceed to sequencing (Agilent Bioanalyzer). Total of 185 cells passed the RNA quality test and proceeded to sequencing. Sequencing with the Illumina HiSeq2500 was carried out with single-end, 100 base-reads, and an average per cell read depth of 2.6 million.

The breakdown of cells captured and analysed in single-cell RNAseq are depicted in Supplementary Table 1. For some groups we compare Postn lineage-traced cells (eGFP^+^) with eGFP-negative cells although a small number of these negative lineage-traced cells were Postn mRNA expressing, reflecting the inefficiency of the Cre-loxP system.

### Bioinformatics of RNAseq

RNAseq analysis was performed as described previously^54^,^55^. Briefly, quality assurance analysis was performed on all the cells using a heat map of the top 12,043 genes expressed that had greater than five TPM in at least one cell, ranked by the average expression of these genes across all samples from highest to lowest. This heat map showed us evident trends of transcripts where activated cell populations had significantly more transcripts compared with the resident quiescent cell populations. However only a very few cells were identified with poor library depth based on the library genes which were used for analysis but not presented in the figures. Genes with >5 TPM in at least one cell from the 12,043 total gene coverage were analysed for differential expression and to identify different cell types or the principle groups of cells that were present in the populations. Differentially expressed genes and cell classification was carried out using both log2 (TPM+1) normalized or further median-normalized expression values. Genes that were significantly different between activated and non-activated were used to classify the cells and identify other genes that were different within the activated or non-activated cells with hierarchical clustering. Gene lists of relative enrichments for various functional associations were determined using ToppGene. The RNAseq data were uploaded to GEO database (GSE83337).

### Western blots

Western blotting was performed as described previously^56^. Briefly, hearts were homogenized in RIPA buffer (50 mM Tris-HCl, 1 mM EDTA, 1 mM EGTA, 150 mM NaCl, 1% NP-40) containing protease inhibitor cocktail (Sigma-Aldrich P8340) with a Dounce homogenizer. Forty micrograms of protein per sample were resolved on 10% SDS–polyacrylamide gel electrophoresis gels, transferred onto PVDF membranes, immunoblotted with antibodies for periostin (Novus BiologicalsNBP1-30042); Cre (Novagen 69050-3); GFP (Novus Biologicals NB600-310) at a 1:800 dilution and GAPDH (Fitzgerald 10R-G109a) at a 1:20,000 dilution, and then incubated with the appropriate alkaline phosphate-linked secondary antibody. PVDF membranes were visualized by enhanced chemifluorescence (Amersham). Uncropped versions of the scans are presented in Supplementary Fig. 12.

### Cell isolation and sorting for qPCR

Adult cardiomyocytes were isolated by removal of beating hearts from anaesthetized mice and cannulated for retrograde perfusion with modified Tyrode solution (NaCl 120 mM, KCl 14.7 mM, KH_2_PO_4_ 0.6 mM, Na_2_HPO_4_ 0.6 mM, MgSO_4_ 1.2 mM, HEPES 10 mM, NaHCO_3_ 4.6 mM,taurine 30 mM,glucose 5.5 mM, butanedionemonoxime (BDM) 10 mM, pH7.4) supplemented with LiberaseTH (Roche)^44^. After perfusion, hearts were disassociated into individual cardiomyocytes and aggregated by two serial centrifugations at 10*g* for 5 min at 4 °C and the non-cardiomyocyte cell fraction was collected after a final centrifugation at 500 g for 10 min at 4 °C. Endothelial (CD31+) and myeloid (CD45^+^) cell fractions were sorted out with a Magnetic Cell Isolation and Cell Separation kit (Miltenyi Biotec) per manufacturer’s instructions with antibodies against CD31 (Miltenyi Biotec 130-097-418) and CD45 (Miltenyi Biotec 130-052-301) using manufacturer’s recommended dilutions.

### Quantitative real-time PCR

RNA was isolated from sorted cells using the RNeasy Mini Kit (QIAgen) and cDNA synthesized using the Verso cDNA Synthesis Kit (Thermo Scientific) according to the manufacturer’s protocols. Quantitative real-time PCR (qRT-PCR) was performed on a BioRad CFX Connect Real-Time System using BioRad SsoAdvanced Universal SYBR Green Supermix and primers specific for Col1a1 (Col1a1 qPCR-Fw 5′-GCCAAGAAGACATCCCTGAAG-3′ and Col1a1 qPCR-Rev 5′-TGTGGCAGATACAGATCAAGC-3′) or 18S as described previously^57^. Data were generated using the standard curve and normalized to 18S expression.

### Statistics

For studies involving cardiac injury such as MI, group sizes were determined based on previously observed post-operative mortality rates for this procedure. No experimental animals were excluded in any of the analyses. For flow cytometry experiments and direct counting of cells in histological sections two-group comparisons were performed using Student’s two-tailed *t*-test, with *P*<0.05 considered statistically significant. All error bars throughout the figures are s.e.m. and all represented data are averages. When representative FACS plots or immunohistological images are shown, at least three independent samples were analysed from separate mice. Animal numbers and sample sizes reflected the minimal number needed for statistical significance based on power analysis and prior experience. No data were excluded from any of the experiments, and randomization and blinding were not performed because it was not appropriate for the types of animal groups used here, or the types of comparisons used between groups.

### Data availability

RNA sequencing data generated in this manuscript that support the findings of this study have been deposited in GEO (Gene Expression Ominbus) of NCBI under accession code GSE83337, which are also listed, in part, in the first three Supplemental Data sets as Excel spreadsheets. All other relevant data are available upon request from the authors.

## Additional information

**How to cite this article:** Kanisicak, O. *et al*. Genetic lineage tracing defines myofibroblast origin and function in the injured heart. *Nat. Commun.* 7:12260 doi: 10.1038/ncomms12260 (2016).

## Supplementary Material

Supplementary Information Supplementary Figures 1-12 and Supplementary Table 1

Supplementary Data 1 Gene expression enrichment and GO term annotation for Postn-lineage traced cells versus CD31-CD45-Postn- cells from MI injured heart or CD31-CD45- from uninjured heart (populations compared in Fig3)

Supplementary Data 2 The average gene expression levels shown in the heat map of the 1048 genes with >5 TPM in at least one cell and differentially expressed between cell population groups in figure 7f with 5% FDR

Supplementary Data 3 Gene expression changes and GO annotation between Postn+ lineage traced cells in Ang/PE actively injured hearts versus hearts 2 weeks after Ang/PE
