# Supplementary material for: Genetic lineage tracing defines myofibroblast origin and function in the injured heart
Source: Nat Commun. 2016 Jul 22;7:12260. doi: 10.1038/ncomms12260 (PMC5512625; doi:10.1038/ncomms12260)
Supplement: Supplementary Information — Supplementary Figures 1-12 and Supplementary Table 1 [file ncomms12260-s1.pdf]

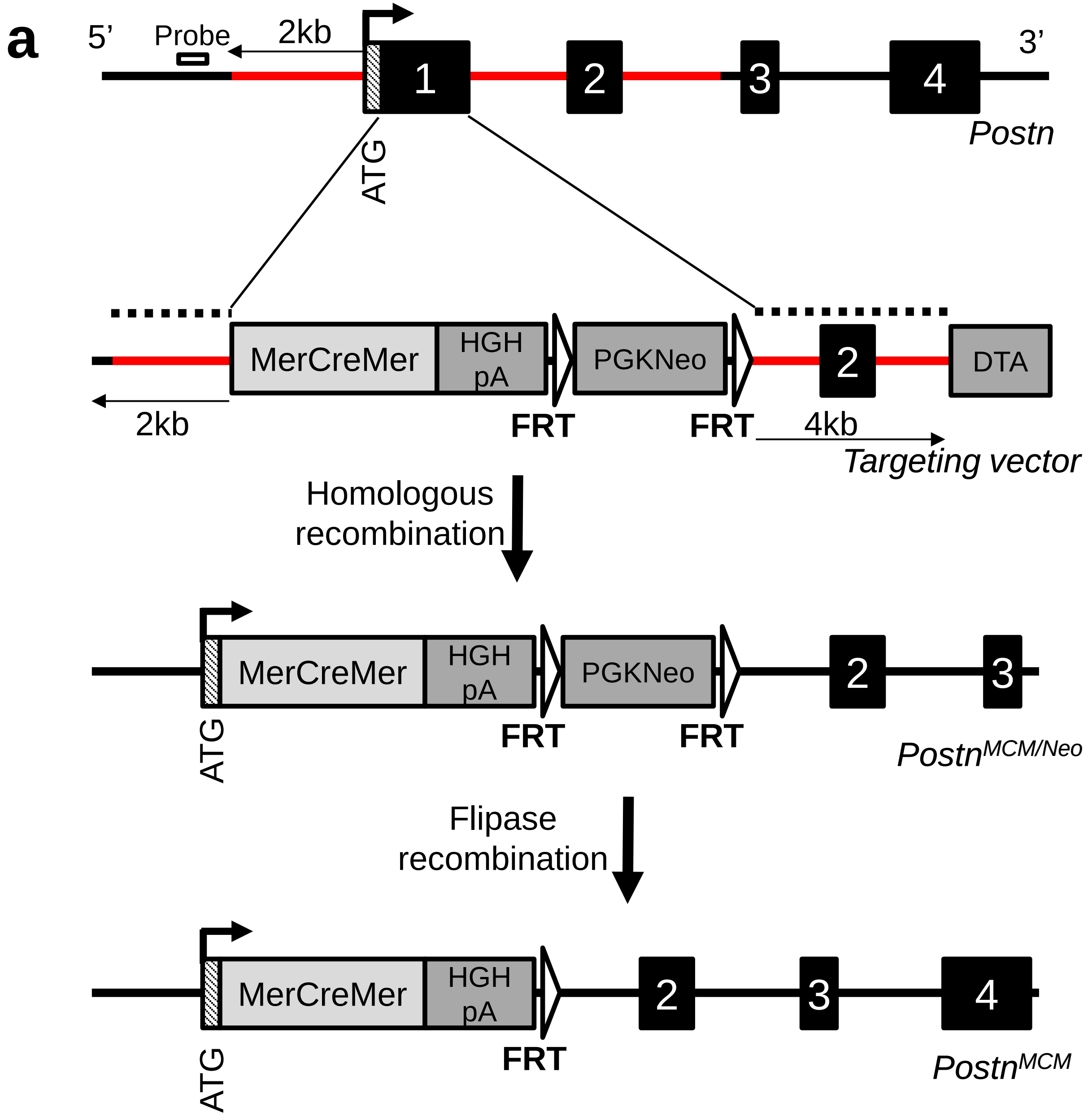

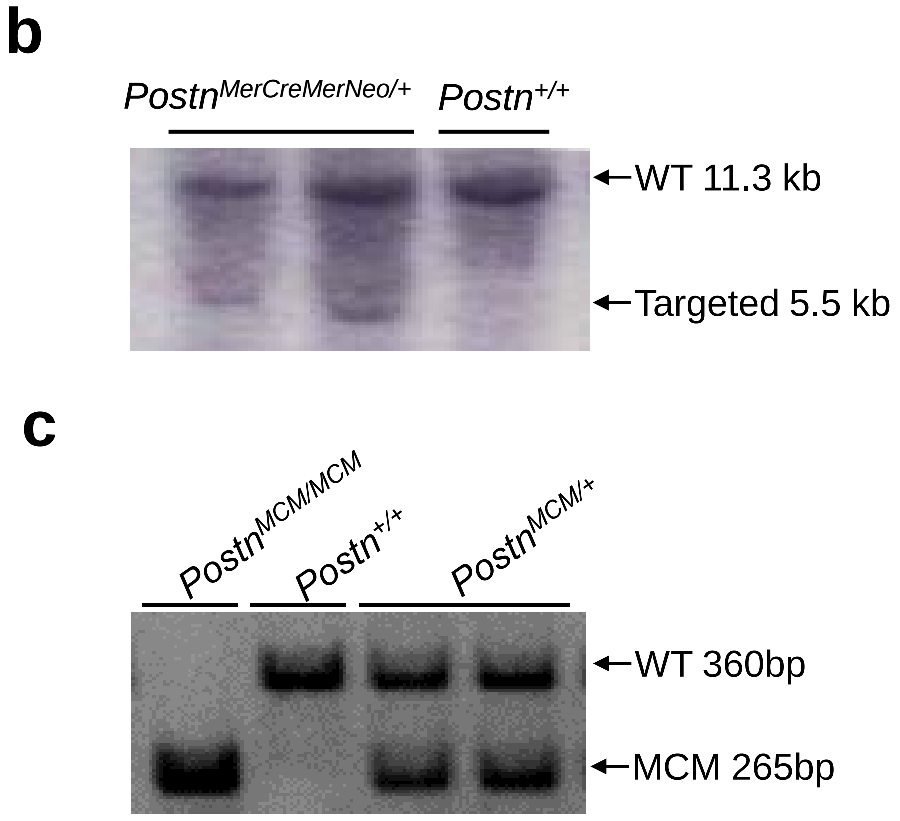

Supplementary Figure 1. Generation of the *Postn* knock-in mouse line containing the MerCreMer cDNA cassette (*Postn*^{MCM}). (**a**) Schematic of the *Postn* genetic locus and targeting vector used to make the MerCreMer cDNA knock-in targeted embryonic stem and Flipase recombined mice to produce the final genetic configuration in mice. The cDNA encoding the MerCreMer fusion protein was cloned in-frame with the *Postn* ATG start site of exon 1. Germline transmitting male chimeras were crossed with *Rosa26*-Flpe females to delete the neomycin cassette at the designed FRT sites to produce heterozygous *Postn*^{MCM} mice that were used here. The position of the Southern probe is shown. (**b**) Southern blot of the *Postn* genetic locus showing correctly targeted embryonic stem cells after BamHI digestion producing fragments of the predicted size. (**c**) DNA fragment migration in an agarose cell showing the correct PCR fragments from the *Postn* locus for the genotype of mice shown (genomic DNA was used for PCR and Southern blotting).

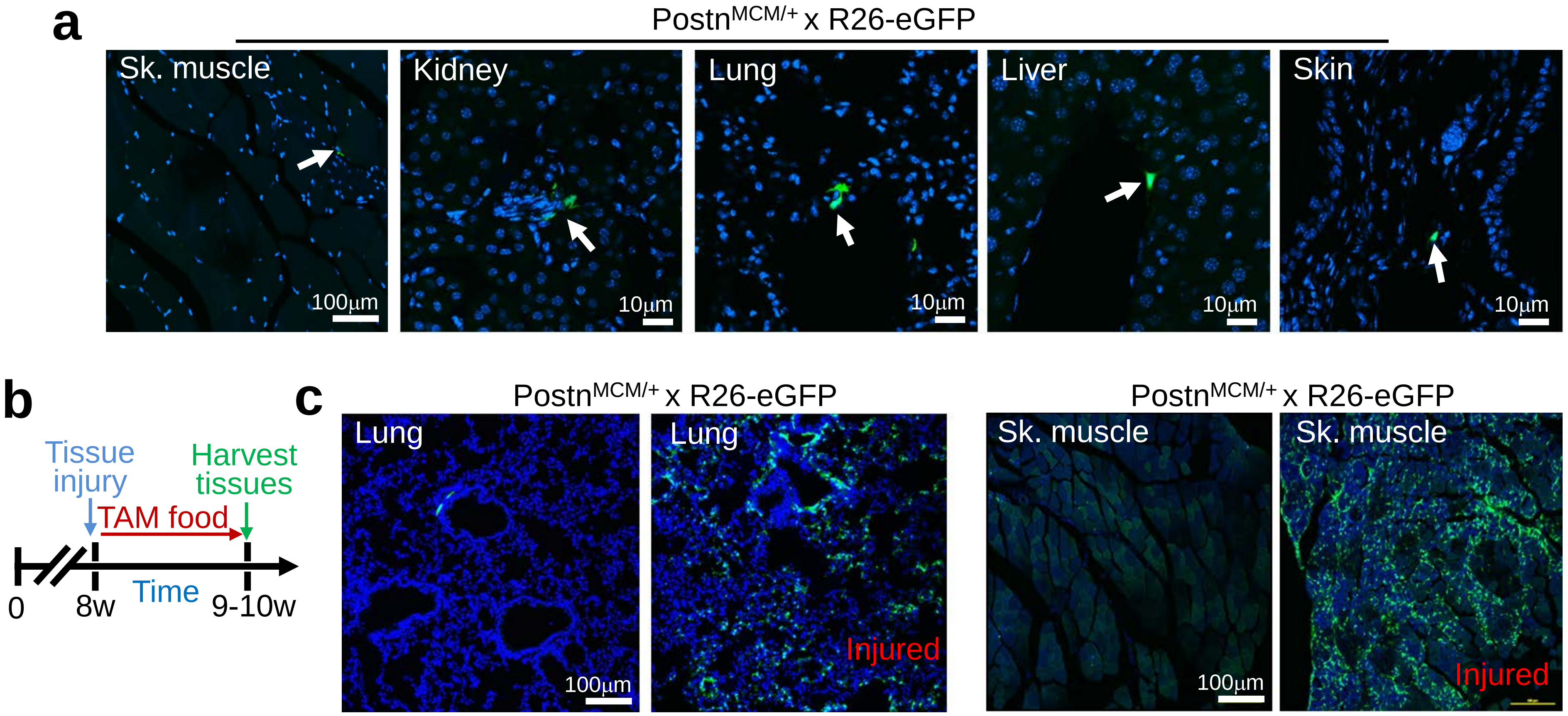

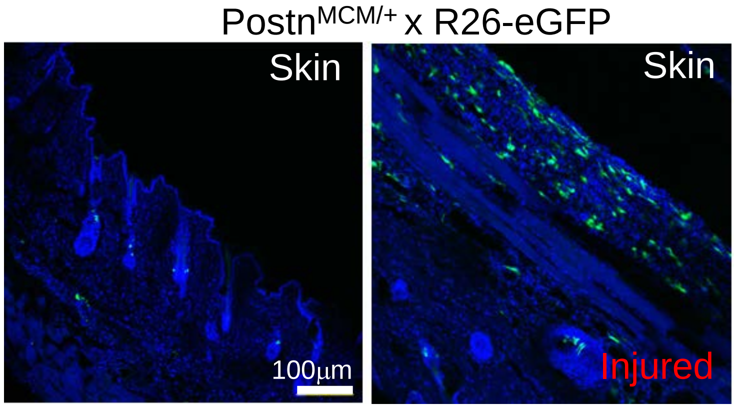

Supplementary Figure 2. *Postn*^{MCM} allele is silent in uninjured adult tissues but is strongly induced after injury. (**a**) Representative histological sections from the indicated tissues showing almost no labeling over 8 weeks of tamoxifen (weeks 8 to 16 weeks of age) in *Postn*$^{MCM/+}$;R26-eGFP mice without injury. The arrows show a single rare cell labeled in each tissue (n=4 mice). (**b**) Experimental scheme in adult *Postn*$^{MCM/+}$;R26-eGFP mice to label cells upon injury in vivo with 1-2 weeks of tamoxifen administration. Nuclei are shown in blue. (**c**) Representative histological sections from the 3 tissues shown that were either uninjured or injured (n=3 mice for each condition). Abundant interstitial cells were labeled (green, eGFP^{+}) following injury. Lung injury was accomplished by 14 days after TAC surgery, which increases pressure to the lungs. Skeletal muscle injury was assessed 7 days after cardiotoxin intramuscular injection, and skin injury was assessed 7 days after punch biopsy wounding, and the border zone was analyzed.

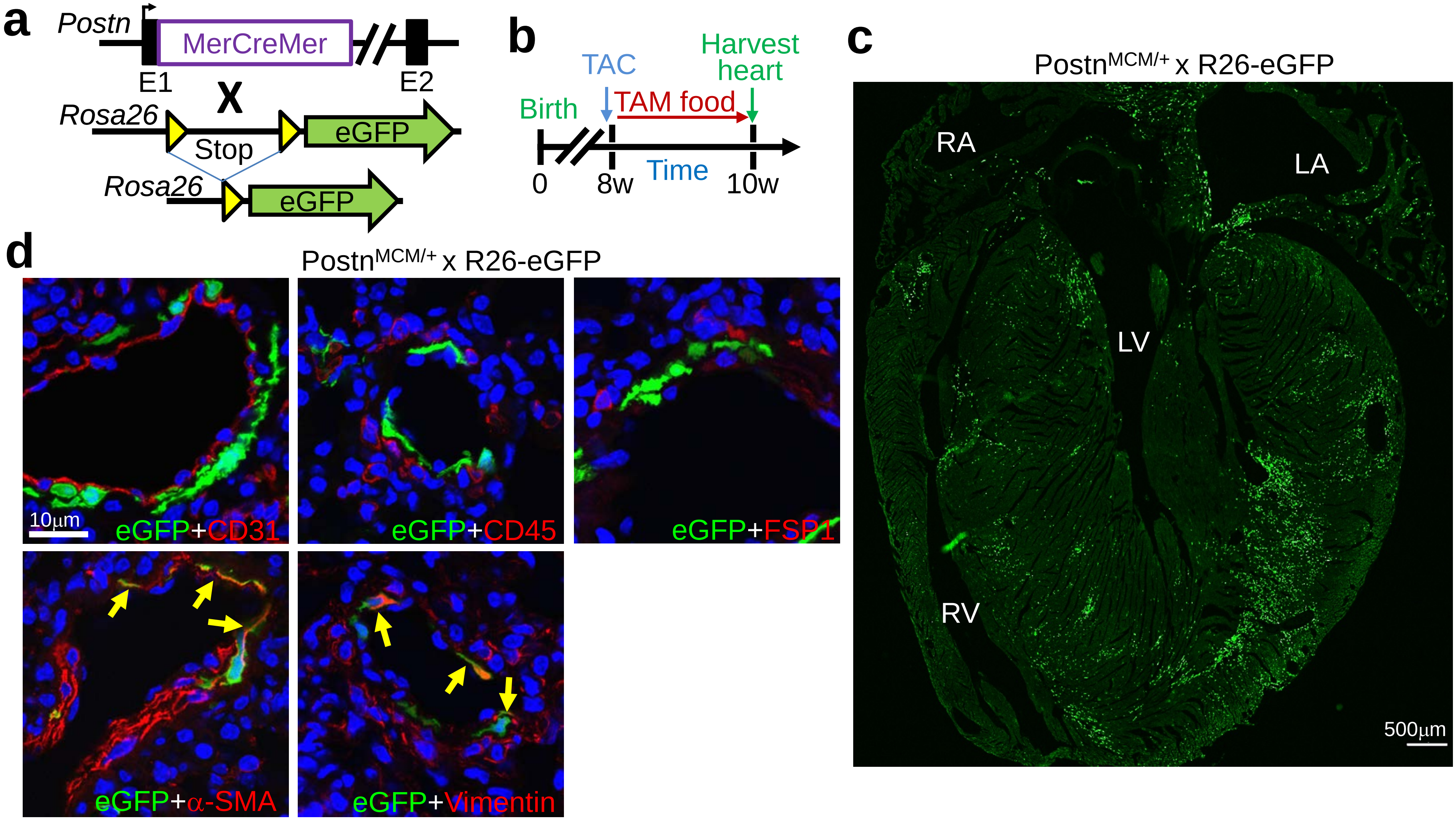

Supplementary Figure 3. The *Postn*^{MCM} allele labels myofibroblasts with pressure overload injury. (**a**) Schematic of the *Postn*^{MCM} mouse crossed with a *Rosa26-eGFP* reporter mouse (R26-eGFP) for lineage tracing. (**b**) Experimental scheme to lineage trace periostin expressing cells in vivo for 2 weeks with tamoxifen treatment immediately after TAC surgery to induce pressure overload. (**c**) Representative histological section through the entire mouse heart in *Postn*$^{MCM/+}$;R26-eGFP mice showing eGFP$^+$ (periostin lineage-traced) interstitial cells (myofibroblasts) after TAC. Without TAC stimulation almost no eGFP$^+$ cells were observed with 2 weeks of control tamoxifen administration (not shown). The eGFP$^+$ periostin lineage-traced cells were both interstitial throughout the heart and also surrounding vessels (perivascular). Abbrev; LV, left ventricle; LA, left atria; RV, right ventricle; RA, right atria. (**d**) Representative histological heart sections containing a vessel showing eGFP$^+$ (periostin lineage-traced) cells co-stained with antibodies for the depicted markers in red. Yellow arrows indicate co-labeling of eGFP and the cell markers depicted. Periostin$^+$ cells after TAC stimulation had the same profile as after MI injury to the left ventricle, such that the vast majority of eGFP$^+$ cells were co-stained with vimentin and αSMA, but almost none stained for CD45 (immune cells), CD31 (endothelial) or FSP1 (immune cells). At least 3 hearts each were analyzed in this manner after TAC stimulation.

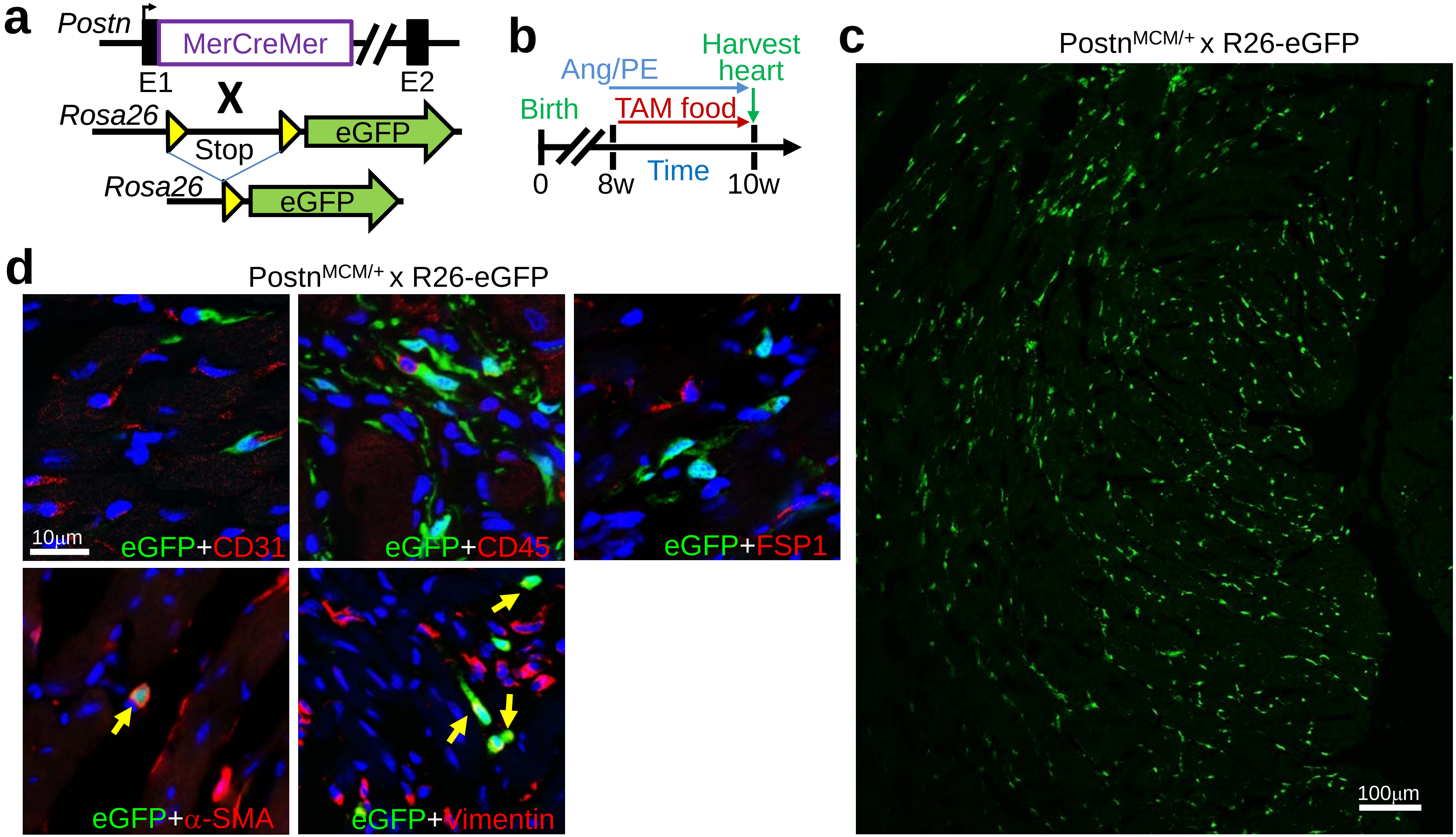

Supplementary Figure 4. The *Postn*^{MCM} allele labels myofibroblasts after Ang/PE stimulation to the heart. (**a**) Schematic of the *Postn*^{MCM} mouse crossed with a *Rosa26-eGFP* reporter mouse (R26-eGFP) for lineage tracing. (**b**) Experimental scheme to lineage trace periostin expressing cells in vivo for 2 weeks with tamoxifen treatment along with Ang/PE agonist infusion to induce cardiac fibrosis. (**c**) Representative histological section through approximately 1/4th of the mouse left ventricle in *Postn*$^{MCM/+}$;R26-eGFP mice, which showed a large induction of eGFP^{+} (periostin lineage-traced) interstitial cells (myofibroblasts). Without Ang/PE stimulation almost no eGFP^{+} cells were observed with 2 weeks of tamoxifen administration (not shown). The eGFP^{+} periostin lineage traced cells were primarily interstitial and distributed throughout all 4 chambers of the heart. (**d**) Representative histological heart sections showing eGFP^{+} (periostin lineage traced) cells co-stained with antibodies for the depicted markers in red. Yellow arrows indicate co-labeling of eGFP and the cell markers depicted. Periostin lineage traced cells after Ang/PE had the same profile as after MI injury to the left ventricle, such that the vast majority of eGFP^{+} cells were co-stained with vimentin and αSMA, but almost none stained for CD45 (immune cells), CD31 (endothelial) or FSP1 (immune cells). At least 3 hearts each were analyzed in this manner after Ang/PE stimulation to the heart.

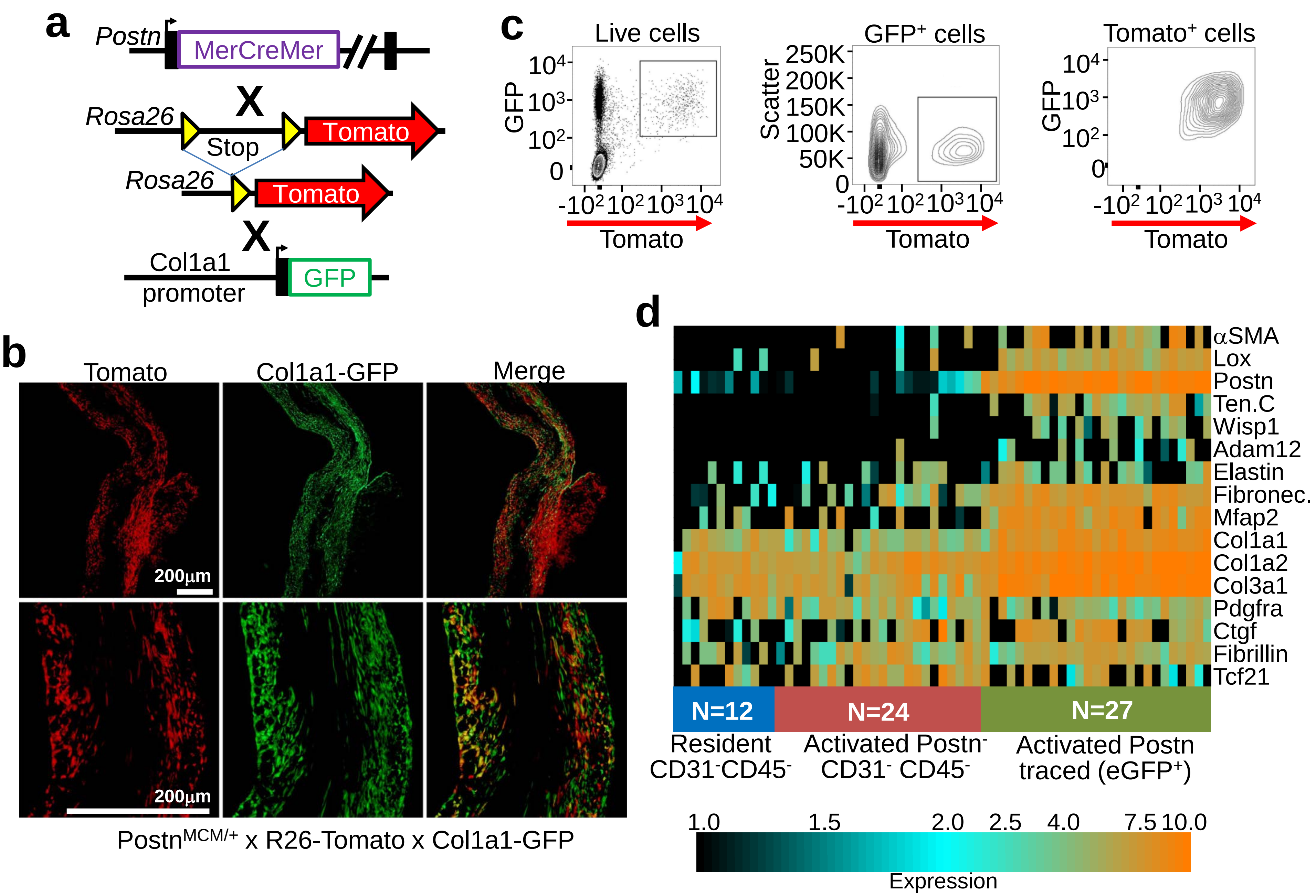
a
Postn
MerCreMer
X
Rosa26
Stop
Tomato
Rosa26
Tomato
X
Col1a1
promoter
GFP
b
Tomato
Col1a1-GFP
Merge
200µm
200µm
PostnMCM/+ x R26-Tomato x Col1a1-GFP
c
Live cells
GFP
Tomato
GFP+ cells
Scatter
250K
200K
150K
100K
50K
0
Tomato
Tomato+ cells
GFP
Tomato
d
αSMA
Lox
Postn
Ten.C
Wisp1
Adam12
Elastin
Fibronec.
Mfap2
Col1a1
Col1a2
Col3a1
Pdgfra
Ctgf
Fibrillin
Tcf21
N=12
N=24
N=27
Resident CD31-CD45-
Activated Postn- CD31- CD45-
Activated Postn traced (eGFP+)
1.0
1.5
2.0
2.5
4.0
7.5
10.0
Expression

Supplementary Figure 5. Col1a1-GFP mouse line labels all periostin lineage-traced myofibroblasts. (**a**) Schematic of the *Postn*^{MCM} mouse crossed with *Rosa26*-Tomato reporter mouse that was also crossed to contain the collagen1a1-GFP reporter transgene. (**b**) Representative cardiac histological sections through the MI region from hearts of *Postn*$^{MCM/+}$;R26-Tomato;Col1a1-GFP mice taken 7 days after MI injury. The images show collagen1a1-GFP expression in green and periostin lineage-traced cells in red. Greater than 95% of the Tomato^{+} cells expressed the collagen1a1-GFP transgene. (**c**) FACS analysis of enzymatically isolated interstitial cells showing almost 100% co-labelling of periostin lineage-traced cells and Col1a1-GFP positivity in MI injured hearts from *Postn*$^{MCM/+}$;R26-Tomato;Col1a1-GFP mice. These cells were also isolated 7 days after MI injury. Total of 3 mice were used to gather data from *Postn*$^{MCM/+}$;R26-Tomato;Col1a1-GFP mice and greater than 5 non-sequential sections from 2 infarcted regions were analyzed for histology. (**d**) Heat map of single cell mRNA sequencing results for each cell used in Figure 3g to generate averaged data for the indicated genes. Gene expression per cell is shown on black-turquoise-orange color scale that depicts Transcripts Per Million (Log2(TPM+1) where Orange: high, Turquoise medium, Black: low). The heat map shows increased levels of myofibroblast genes in activated Postn-traced (eGFP^{+}) cells compared to quiescent or activated non-Postn-traced interstitial cells.

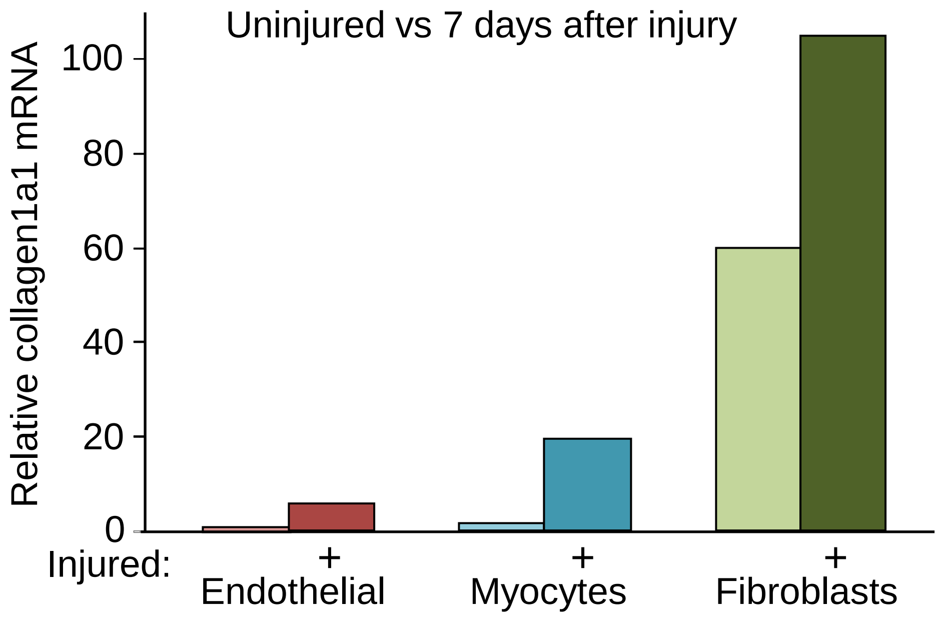

Supplementary Figure 6. Relative collagen1a1 mRNA levels from the 3 major cell types of the heart. The data are qPCR results from the indicated isolated cell types from heart before and after injury. The data show that while both endothelial cells and cardiomyocytes express collagen1a1, the $CD31^{-}$ $CD45^{-}$ fraction that contains fibroblasts expresses the highest levels. Moreover, upon injury collagen expression is induced in all three cell types, although fibroblasts remain the greatest contributor . Cells from at least 2 hearts of each condition were analyzed.

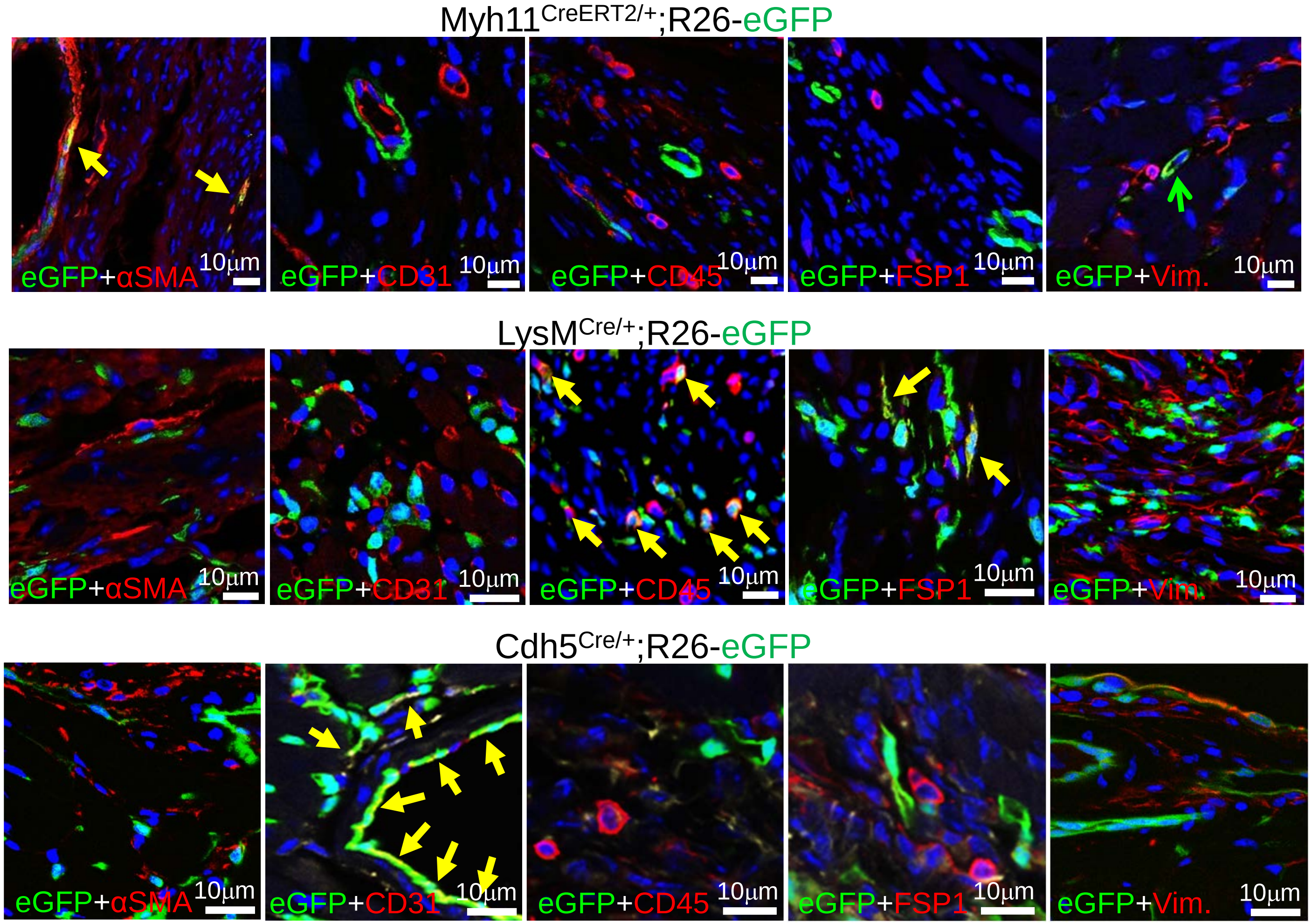

Supplementary Figure 7. Lineage tracing with 3 other Cre alleles in the heart to further define myofibroblast specificity. The images shown are representative of what was used to generate the quantitative data shown in Fig. 5g-i. Immunohistochemistry from heart sections are shown against vimentin, αSMA, CD31, CD45 and FSP1 in red. All green cells were lineage traced from either *LysM*^{Cre}, *Myh11*^{CreERT2}, and *Cdh5*^{Cre} lines, crossed with the *Rosa26*-eGFP reporter line. At least 3 hearts each were analyzed with over 200 cells quantified for each of the indicated genotypes. The yellow arrows show co-staining between eGFP^{+} cells and the indicated marker detected with antibody.

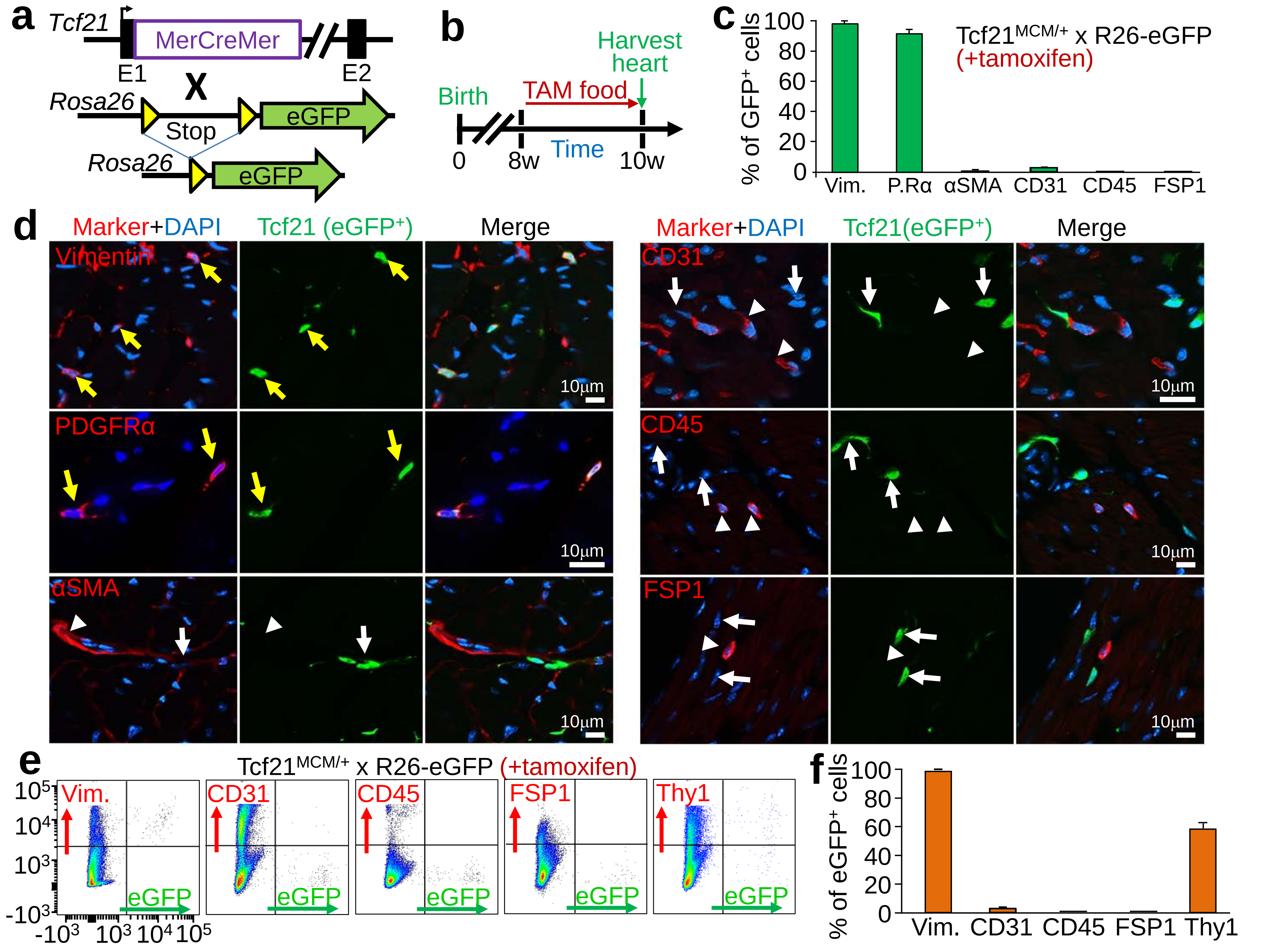

a
Tcf21
MerCreMer
E1
E2
X
Rosa26
Stop
eGFP
Rosa26
eGFP
b
Harvest heart
Birth
TAM food
0
8w
Time
10w
c
% of GFP+ cells
100
80
60
40
20
0
Vim.
P.Rα
αSMA
CD31
CD45
FSP1
Tcf21MCM/+ x R26-eGFP
(+tamoxifen)
d
Marker+DAPI
Tcf21 (eGFP+)
Merge
Vimentin
PDGFRα
αSMA
CD31
CD45
FSP1
10μm
e
Tcf21MCM/+ x R26-eGFP (+tamoxifen)
Vim.
CD31
CD45
FSP1
Thy1
eGFP
10^5
10^4
10^3
-10^3
-10^3
10^3
10^4
10^5
f
% of eGFP+ cells
100
80
60
40
20
0
Vim.
CD31
CD45
FSP1
Thy1

Supplementary Figure 8. *Tcf21*^{MCM} allele labels resident fibroblasts in the adult heart. (**a**) Schematic of the *Tcf21*^{MCM} allele-containing mouse crossed with a *Rosa26*-eGFP reporter mouse (R26-eGFP) for lineage tracing. (**b**) Experimental scheme to label Tcf21 expressing cells *in vivo* for 2 weeks in the adult heart with tamoxifen treatment. (**c**) Quantification of co-labeling of Tcf21 lineage-traced (eGFP^{+}) cells with the indicated cell markers from immunohistochemical processed heart sections (n=3 hearts, >20 sections each were quantified). (**d**) Representative immunohistochemical images for eGFP cellular expression (green) for Tcf21 lineage traced cells and co-staining for vimentin, PDGFRα, αSMA, CD31, CD45 or FSP1 in red. The yellow arrows show co-staining, the white arrows show eGFP^{+} only, and the white arrow heads show marker expression only without eGFP^{+}. (**e**) Representative flow cytometry plots of isolated Tcf21 lineage-traced (eGFP^{+}) cells (rightward scatter) against the cell markers depicted (upwards scatter). (**f**) Quantification of the flow cytometry analysis (n=4 hearts digested and analyzed). All error bars represent s.e.m.

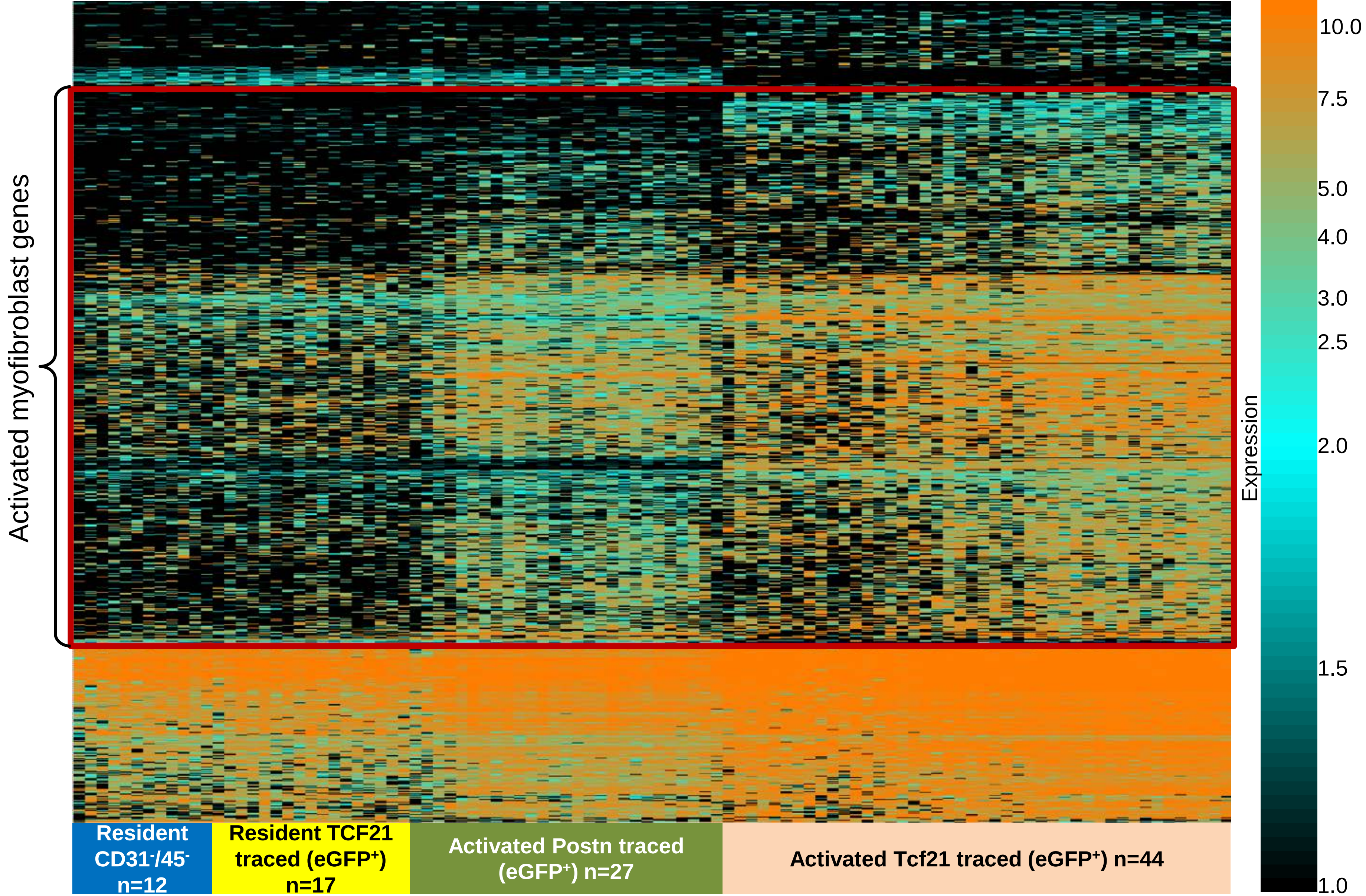

Supplementary Figure 9. Heat map of differentially expressed genes for each single cell used in Figure 7e. Hierarchical clustering of resident CD31$^-$CD45$^-$, resident Tcf21-traced, activated Postn-traced and activated Tcf21-traced was performed to analyze gene expression between groups. Gene expression per cell is shown on black-turquoise-orange color scale that depicts Transcripts Per Million (Log2(TPM+1) where (Orange: high, Turquoise medium, Black: low). The heat map shows expression pattern differences of the 1048 genes (listed in Supplementary Data 2) from the 12,043 genes with >5 TPM in at least one cell and differentially expressed between classes of 5% FDR. Myofibroblast specific genes were upregulated in activated Postn$^+$ and activated Tcf21$^+$ fibroblasts.

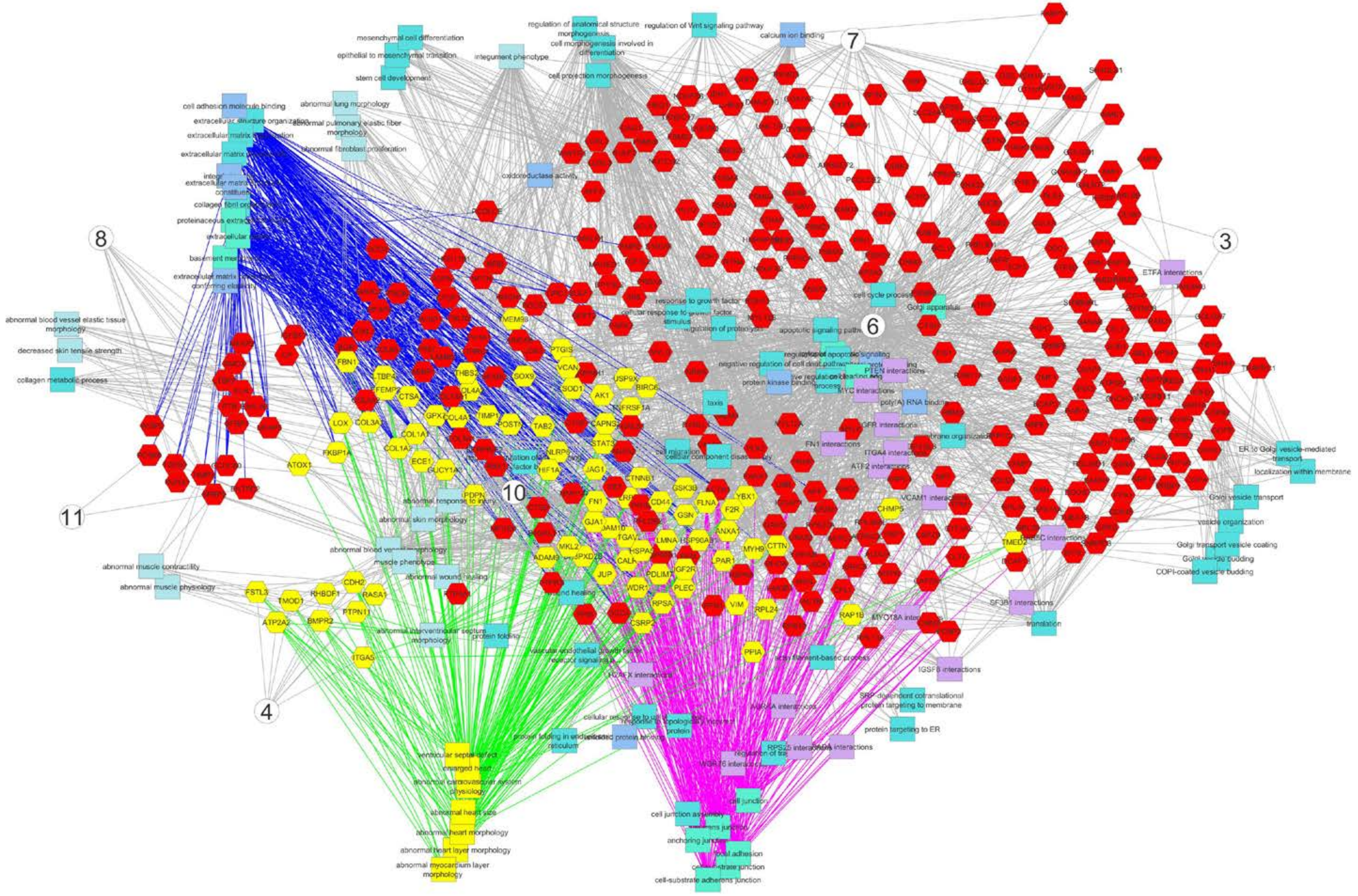

Supplementary Figure 10. Gene network analysis of the 1048 differentially expressed genes shown in Supplementary Figure 9 and Supplementary Data 2. The analysis revealed 11 subgroups of genes differentially regulated in activated Postn-traced fibroblasts. Many of these gene clusters were involved in wound healing, extracellular matrix component production, mesenchymal cell differentiation, cell adhesion and fibrotic disease states.

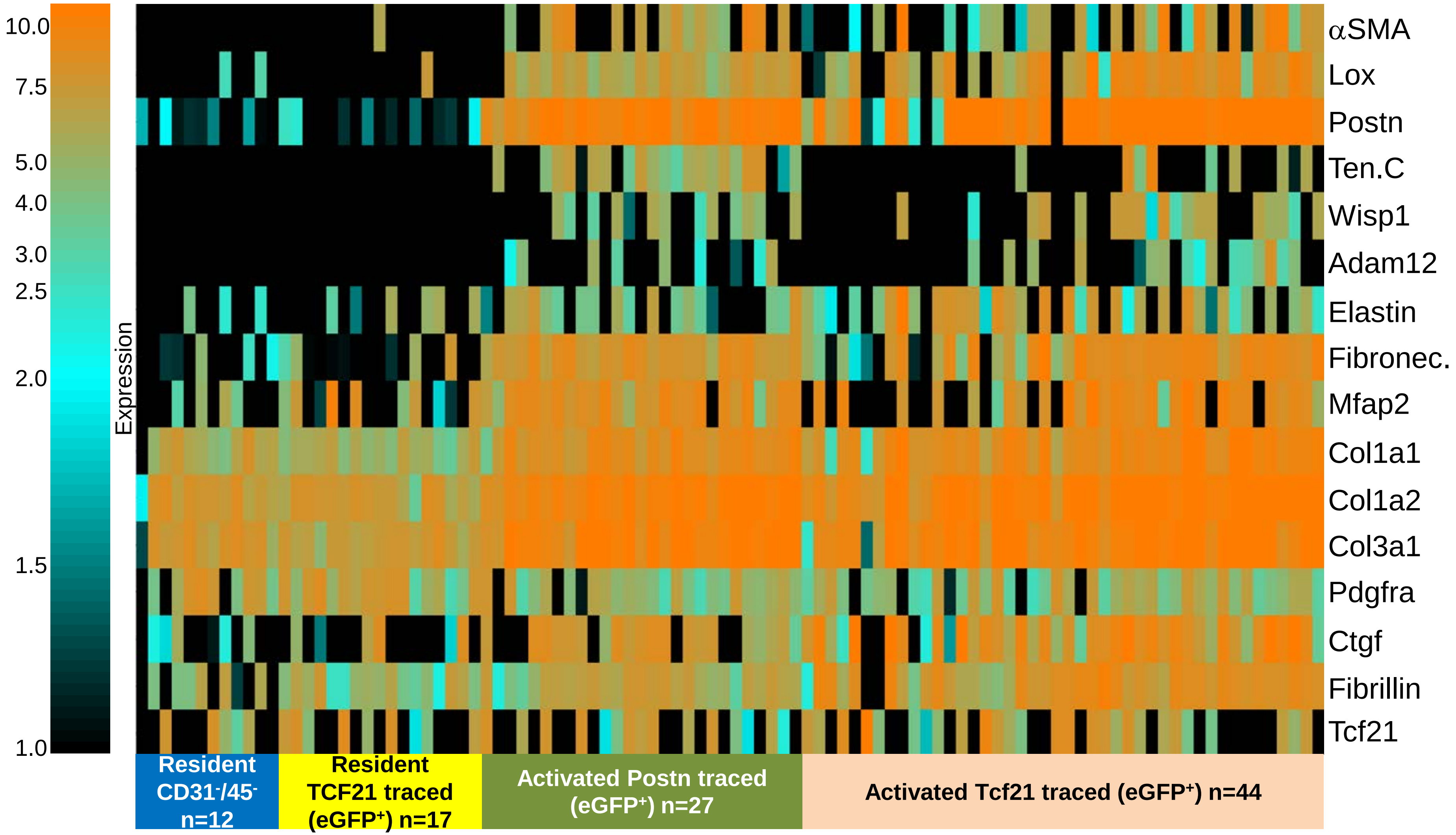

Supplementary Figure 11. Heat map of single cell RNA sequencing results for the depicted genes for each single cell used in Figure 7f, which depicts the averaged data for the cells in each group. Gene expression per cell is shown on black-turquoise-orange color scale that depicts Transcripts Per Million (Log2(TPM+1) where Orange: high, Turquoise medium, Black: low). The heat map shows increased levels of gene expression in activated Postn-traced and Tcf21-traced cells for the 16 myofibroblast related genes.

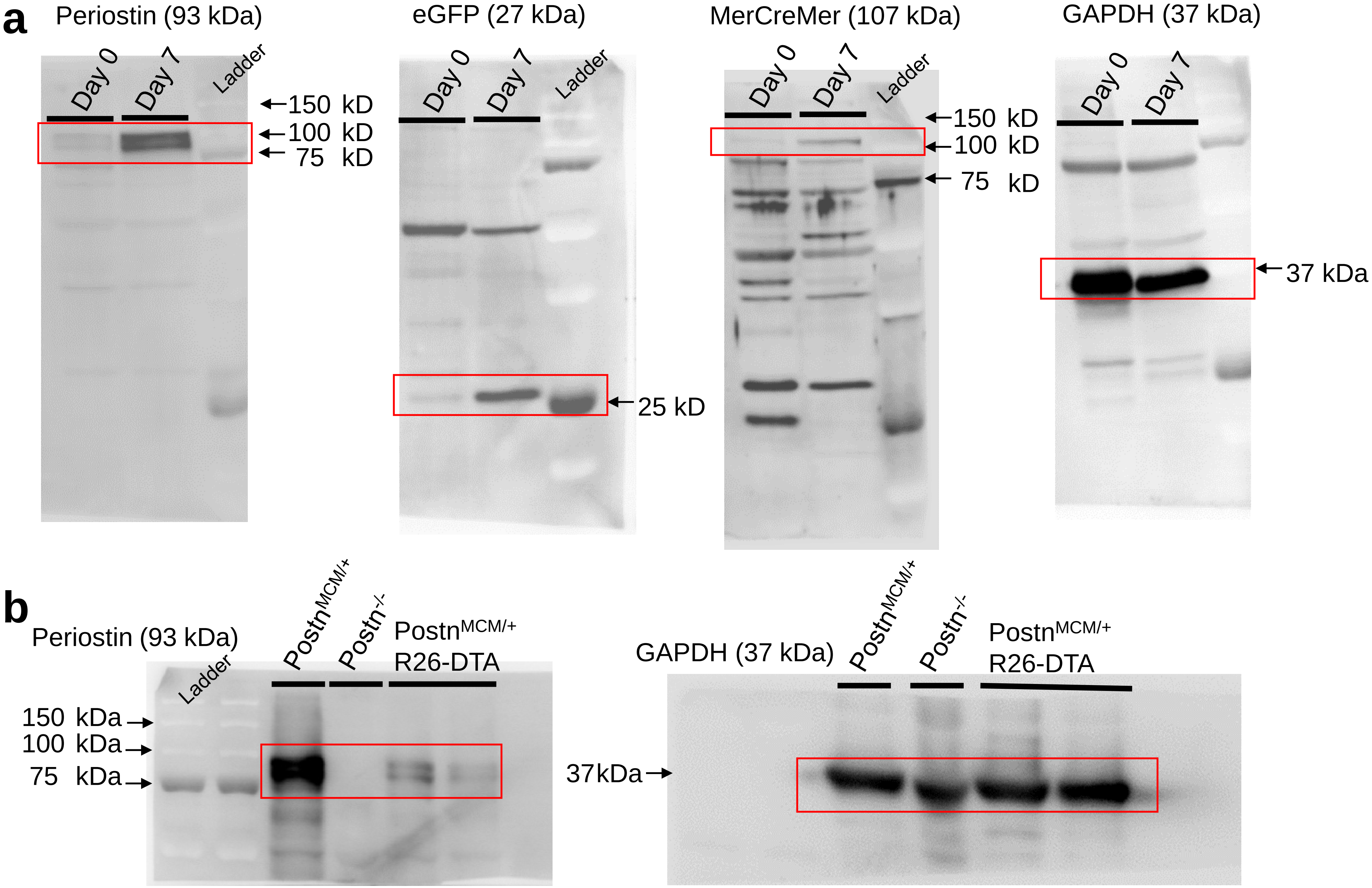

Supplementary Figure 12. uncropped scans of all western blots used in the manuscript. (**a**) Blots used in Ffigure 1e showing Periostin, eGFP, MerCreMer and GAPDH. (**b**) Blots used in Figure 3c showing Periostin and GAPDH. Each experiment has been repeated from at least 3 mice pre group.

Supplementary Table 1. The number of cells captured and analyzed in single cell RNAseq per group.

Source	Cell population	Total # of cells*	Postn+**	# of cells used***	Figure ID	Figure tag
SHAM	CD31-CD45-/Tcf21-	13	1	12	F3g,7f,F8c	Resident CD31-CD45-
SHAM	CD31-CD45-/Tcf21+	20	3	17	F7f,F8c	Resident Tcf21+
MI	CD31-CD45-/Postn-	30	3	24	F3g,	Activated Postn- CD31- CD45-
MI	CD31-CD45-/Postn+	27	27	27	F3g, F4d, F7f	Activated Postn traced (eGFP+)
MI	CD31-CD45-/Tcf21-	10	10	0		
MI	CD31-CD45-/Tcf21+	53	44	44	F7f	Activated Tcf21+
MI	CD31-CD45- of the infarct/Non-traced	32	26	0		

* Total number of cells captured and processed for RNA seq from each source.

** Number of cells positive for Postn mRNA expression per group.

*** Number of cells used in generating the figures listed under Figure ID.
